# Breaking the Mold: Electrophilic Hydrophosphanation Emerges

**DOI:** 10.1002/chem.71103

**Published:** 2026-05-14

**Authors:** Víctor Varela‐Izquierdo, Janeth Navarro, Judit Cano‐Asensio, José A. López, Cristina Tejel

**Affiliations:** ^1^ Departamento de Química Inorgánica Facultad de Ciencias Instituto de Síntesis Química y Catálisis Homogénea (ISQCH) CSIC‐Universidad de Zaragoza Zaragoza Spain

**Keywords:** diphosphaenolate, electrophilic activation, hydrophosphanation, P─C bond formation, rhodium

## Abstract

Herein, we report an unprecedented mechanism for the catalytic hydrophosphanation of olefins in which the key step involves an electrophilic hydride attack on the olefin rather than the classical nucleophilic addition of a phosphanido ligand. This mechanistic paradigm redefines the fundamental reactivity of P–H compounds with C═C bonds under catalytic conditions. The dichotomy between electrophilic and nucleophilic pathways has been elucidated through DFT calculations and experimental kinetic isotope effect (KIE) measurements. We also demonstrate that this mechanistic pattern is applicable to both electronically activated and non‐activated olefins, as well as to non‐symmetric olefins. In a parallel finding, we have identified a novel reactivity mode of hydrido‐phosphanido species, in which the phosphanido moiety engages in an intramolecular attack on a tethered olefin to afford a previously unreported diphosphaenolate. This transformation, due to the amphiphilic behavior of complex [Rh(Tp)(PHPh_2_)(H)(PPh_2_)], expands the chemical space accessible through hydrophosphanation‐derived pathways.

## Graphical abstract

Electrophilic hydride attack on the olefin, instead of the classical nucleophilic addition by a phosphanido ligand, is unveiled in rhodium‐catalyzed olefin hydrophosphanation. This hydride‐driven pathway operates for both activated and inactivated olefins, with transition states characterized by elongated Rh─H bonds and nascent C─H bond formation.

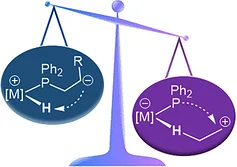

## 1 Introduction

Metal‐catalyzed hydrophosphanation of alkenes/alkynes offers a straightforward and atom‐economical route to a broad range of organophosphorus compounds, which are highly valuable as ligands for transition‐metal complexes, as key intermediates in organic synthesis, and as functional materials with broad commercial applications [1, 2, 3, 4, 5, 6, 7]. This process typically begins with the activation of the P─H bond at a single metal center, which can occur via several mechanistic pathways. Common approaches include the deprotonation of a coordinated phosphane or a proton transfer to an internal base already bound to the metal [3], as well as σ‐bond metathesis for early transition metals [8]. Alternatively, oxidative addition offers a clean and efficient route that does not require previous metal functionalization to give directly hydrido‐phosphanido species [9, 10]. Although such intermediates are ideally suited to participate in catalytic cycles for P−C bond formation, well‐defined hydrido‐phosphanido complexes for late transition metals remain rare, with few structurally characterized examples reported [11, 12, 13, 14, 15, 16].

**SCHEME 1:**
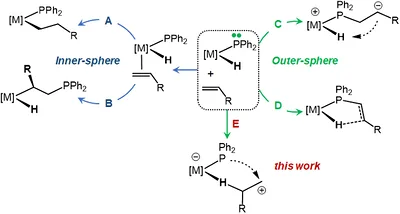
Inner‐ and outer‐sphere pathways proposed for P─C bond formation from hydrido‐phosphanido complexes, illustrated with PHPh_2_ and a generic olefin substrate.

From a mechanistic perspective [17, 18], once the key “H−M−PR_2_” unit is generated, two main pathways have been proposed for P−C bond formation. Namely, coordination of the alkene/alkyne followed by insertion into the M−H or M−P bonds (inner‐sphere mechanisms) and outer‐sphere alternatives, in which the substrate reacts without prior binding to the metal center (Scheme 1). Insertion into the M─H bond (Path A) is generally favored and has been documented in several systems, including Ni‐catalyzed hydrophosphanation of styrenes [19], and alkynes by mono‐ [20] and dinuclear [21] cobalt complexes, as well as in Rh‐mediated ethylene insertion, eventually yielding PPh_2_Et [10].

By contrast, M−P insertion (Path B) is rare, with a single example in platinum chemistry [22]. Computational results show that insertion of alkenes and alkynes into the metal–hydride bond has a lower activation barrier than insertion into the metal–phosphanido bond. This indicates that, across late transition metal systems, reactivity is kinetically biased toward initial hydride involvement [10, 23, 24].

Outer‐sphere mechanisms generally involve nucleophilic attack by the metal‐bound phosphanido lone pair at the electrophilic carbon of the unsaturated substrate to give a zwitterionic intermediate that is subsequently protonated (Path C) [25]. This pathway has been proposed in the hydrophosphanation of formaldehyde with PH_3_ [26], in ruthenium chemistry [27], and thoroughly investigated for the hydrophosphanation of Michael‐type olefins catalyzed by platinum complexes [28, 29]. In such cases, telomerization products are often observed, although their formation can be suppressed by the addition of protic additives [30].

An alternative outer‐sphere process involves a concerted [3+2] cycloaddition between the “H–M–PR_2_” unit and the C═C bond of the substrate (Path D) [31]. This approach, developed by our group in rhodium chemistry, not only circumvents telomerization but also significantly enhances stereoselectivity, typically yielding a single enantiomeric pair due to restricted C─C bond rotation in the transition state (Scheme 1). While both outer‐sphere pathways (Paths C and D) rely on the nucleophilic character of the phosphanido ligand (i.e., nucleophilic hydrophosphanation), the opposite reactivity mode, that is, the electrophilic hydrophosphanation pathway (Path E) remains virtually unexplored.

Herein, we report a remarkably efficient and highly selective rhodium catalyst that enables rapid olefin hydrophosphanation, in which Path E is operative and responsible for the lower activation barriers compared to pathways C and D, as suggested by DFT calculations and KIE measurements. To the best of our knowledge, only one previous example of electrophilic hydrophosphanation has been reported involving phosphenium‐type ligands in molybdenum(0) chemistry (Mo−(PR_2_)^+^) operating through a fundamentally different mechanistic manifold [32].

## 2 Results and Discussion

The catalytic activity of various hydrido‐phosphanido complexes in the hydrophosphanation of olefins with PHPh_2_ was initially assessed using dimethyl fumarate (fum, *trans*‐MeO_2_C─CH═CH─CO_2_Me) as a model substrate. This olefin was selected because the two carbons of the C═C bond are electronically equivalent, ensuring that any observed regioselectivity arises from the different roles of the hydride and phosphanido ligands rather than from intrinsic olefin asymmetry (Table 1).

**TABLE 1:** Catalytic activity of complexes **1**–**5** in the hydrophosphanation of dimethyl fumarate with PHPh_2_.^a^

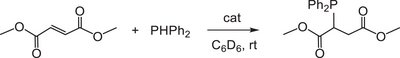				
Entry	Catalyst	Cat (mol%)	Time (min)	Conv. (%)^b^
1a	[Rh(Tp)(PHPh_2_)(H)(PPh_2_)] (**1**)	5	< 5	> 99
1b	[Rh(Tp)(PHPh_2_)(H)(PPh_2_)] (**1**)	**1**	35	> 99
2	[Rh(Tp)(PMe_3_)(H)(PPh_2_)] (**2**)	5	35	> 99
3	[Rh(Tp)(PMe_2_Ph)(H)(PPh_2_)] (**3**)	5	42	> 99
4	[Rh(Tp)(PMePh_2_)(H)(PPh_2_)] (**4**)	5	< 5	> 99
5	[Rh(Tp)(IMes)(PHPh_2_)] (**5**)	5	4320	> 99

^a^ Reaction conditions: PHPh_2_ (0.48 mmol) and alkene (0.48 mmol), catalyst (0.024 mmol, 5% mol) in C_6_D_6_ (V_t_ = 0.7 mL) at 25°C. For catalytic loadings of 1%, the appropriate amount of catalyst was taken from a stock solution, and the total volume was adjusted to 0.7 mL, [PHPh_2_] = [olefin] = 0.46 M (entry 1b).

^b^ Determined by ^1^H NMR spectroscopy. IMes = 1,3‐dimesitylimidazol‐2‐ylidene.

**SCHEME 2:**
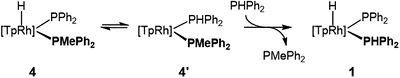
Transformation of complex **4** into **1** under catalytic conditions.

Under standard conditions (5 mol% catalyst in C_6_D_6_ at 25 °C), complex [Rh(Tp)(PHPh_2_)(H)(PPh_2_)] (**1**, Tp = hydrotris(pyrazolyl)borate) [10] rapidly produced the functionalized phosphane Ph_2_P–CH(CO_2_Me)CH_2_(CO_2_Me) (entry 1a). Furthermore, its high efficiency also enabled the reaction to be completed in less than 1 h with just 1% catalyst (entry 1b). The related complexes [Rh(Tp)(PMe_3_)(H)(PPh_2_)] (**2**) [31] and [Rh(Tp)(PMe_2_Ph)(H)(PPh_2_)] (**3**), containing more basic phosphanes, showed similar reaction times to reach completion (entries 2 and 3), but they were less active than complex **1**. The comparable activity of complexes **1** and [Rh(Tp)(PMePh_2_)(H)(PPh_2_)] (**4**, entry 4), which is in equilibrium with its Rh(I) form [Rh(Tp)(PMePh_2_)(PHPh_2_)] (**4′**, Scheme 2) [10], is explained by a facile exchange of PMePh_2_ for PHPh_2_ in species **4′**, forming the active hydrido‐phosphanido complex [Rh(Tp)(PHPh_2_)(H)(PPh_2_)] (**1**) under catalytic conditions. This is supported by the observation of signals for complex **1** and free PMePh_2_ in the ^31^P{^1^H} NMR spectrum at the beginning of the reaction.

In contrast, the rhodium(Ι) complex [Rh(Tp)(IMes)(PHPh_2_)] (**5**, IMes = 1,3‐dimesitylimidazol‐2‐ylidene), for which the corresponding hydrido‐phosphanido rhodium(III) counterpart was not observed in solution, showed very slow catalysis (entry 5). This residual activity is likely due to trace amounts of complex **1**, supporting hydrido‐phosphanido species as the active catalysts.

The enhanced performance of [Rh(Tp)(H)(PHPh_2_)(PPh_2_)] (**1**) compared to [Rh(Tp)(H)(PMe_3_)(PPh_2_)] (**2**) is particularly remarkable. Given that the ancillary PHPh_2_ ligand in **1** is substantially less basic than PMe_3_ in **2**, the phosphanido group in **1** would be expected to exhibit lower nucleophilicity. From a classical S*_N_* ^2^ perspective, in which the reactivity is driven by the lone pair on the phosphanido ligand, complex **2** should therefore be more reactive. Contrary to this expectation, complex **1** demonstrates superior activity. This observation strongly indicates that P−C bond formation does not proceed via a nucleophilic pathway and that the nucleophilicity of the phosphanido ligand is not the determining factor in the reaction mechanism.

Further insight was gained from DFT‐calculated energy profiles for the key P─C bond‐forming step. The reaction of **1** with dimethyl fumarate yields the rhodium(I) complex [Rh(*κ* ^2^‐Tp)(PHPh)(PRPh_2_)] (**6**, R = CH(CO_2_Me)CH_2_CO_2_Me), which lies 5.3 kcal mol^−1^ below the energy of the starting materials (“**1** + fum”), indicating an overall exergonic transformation.

**FIGURE 1:**
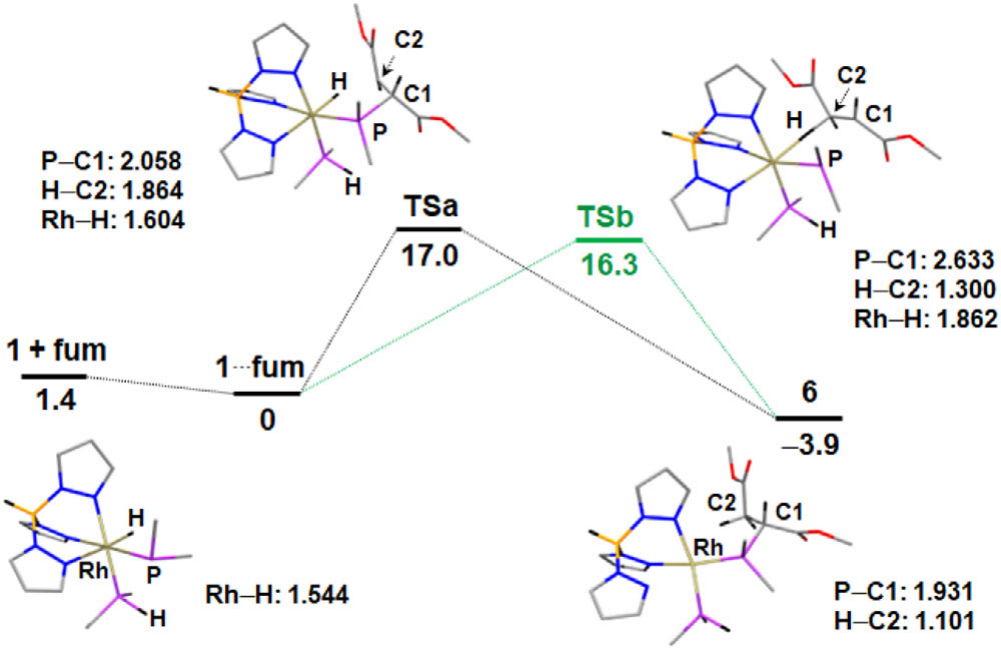
DFT computed Gibbs energy profile for the reaction of [Rh(Tp)(PHPh_2_)(H)(PPh_2_)] (**1**) with dimethyl fumarate. Relative Δ*G*_298_ values and selected bond distances are given in kcal mol^−1^ and Å, respectively. Only the C*^ipso^* of the phenyl groups is shown for clarity. Color code: Rh (yellow), O (red), N (blue), C (gray), B (orange), and H (black).

As depicted in Figure 1, the phosphanido ligand can attack the C1 carbon of the olefin via transition state **TSa** (black pathway), with an activation barrier of 17.0 kcal mol^−1^ relative to the adduct “**1** ^…^fum.” Interestingly, a second, lower‐energy transition state, **TSb** (green pathway), was also located. In this case, C−H bond formation occurs first, as the Rh─H bond elongates from 1.544 Å (in **1**) to 1.862 Å, indicating an incipient hydride transfer to the C2 carbon of the alkene.

These results provide direct mechanistic evidence that the reaction can proceed via two distinct pathways: either a conventional nucleophilic attack by the phosphanido group to the free olefin (**TSa**, Path D in Scheme 1) or a hydride transfer from rhodium to the substrate (**TSb**, Path E). The **TSb,** lower in energy than **TSa**, supports the electrophilic hydrophosphanation mechanism over the nucleophilic one. From an outer‐sphere perspective involving the “H─Rh─P” fragment and the olefin C═C bond, **TSa** arises from interaction of the phosphanido lone pair with the olefin π‐antibonding orbital, whereas **TSb** involves donation from the olefin π‐bond to an empty orbital of the hydrido ligand. Consistent with this interpretation, an intermolecular competition experiment [33] gives a kinetic isotope effect of 3.0, providing strong evidence for substantial Rh─H bond rupture in the rate‐determining transition state (Figure S20). Moreover, we reported a quite lower KIE (1.27) for the slower catalyst [Rh(Tp)(PMe_3_)(H)(PPh_2_)] (**2**), which operates through the initial P−C bond formation (nucleophilic path).

Both transition states exhibit strictly planar “Rh─P─C1─C2─H” frameworks, with maximum deviations from planarity of 0.027 and 0.068 Å for **TSa** and **TSb**, respectively. Furthermore, the geometries around C2 in **TSa** and C1 in **TSb** are nearly trigonal‐planar, with angle sums (Σ°) of 355.7° and 357.6°, respectively. Notably, **TSa** appears to benefit from an additional stabilizing interaction between the electrophilic Rh─H fragment and the lone pair located in the p_z_ orbital of C2. Conversely, in **TSb**, the lone pair on the nucleophilic phosphanido ligand engages with the vacant p_z_ orbital of C1, offering a distinct and complementary mode of stabilization.

**SCHEME 3:**
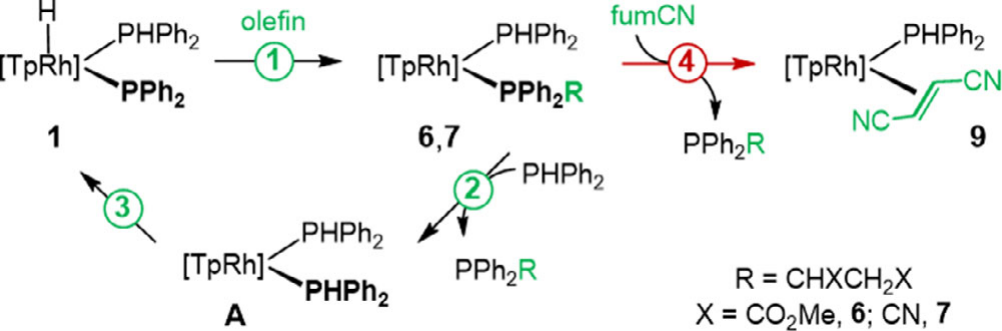
Catalytic cycle for olefin hydrophosphanation with PHPh_2_ (steps 1–3) and catalyst deactivation via step 4. Olefin = dimethyl fumarate, fumaronitrile.

Once complex **6** is formed via step‐1, closing of the catalytic cycle is easily achieved by phosphane exchange (step‐2) followed by P−H bond activation in intermediate [Rh(*κ* ^2^‐Tp)(PHPh_2_)_2_] (**A**, Scheme 3).

The reaction of **1** with fumaronitrile (fumCN, *trans*‐NC−CH═CH─CN) to form [Rh(*κ* ^2^‐Tp)(PHPh)(PRPh_2_)] (**7**, R = CH(CN)CH_2_CN) was also investigated by DFT calculations. A single transition state (**TSb’**) was located, featuring an elongated Rh−H bond (1.887 Å) and a forming C−H bond (1.267 Å, Figure S1). **TSb’** lies 17.6 kcal mol^−^^1^ above the energy of the “**1** ^…^fumCN” adduct, slightly higher than the corresponding barrier for fumarate (16.3 kcal mol^−^^1^). Despite this small difference, the hydrophosphanation of fumCN with PHPh_2_ catalyzed by **1** (5% mol‐cat) slows down significantly after the first catalytic cycle. At this stage, the ^3^ ^1^P{^1^H} NMR spectrum shows [Rh(Tp)(PHPh_2_)(fumCN)] (**9**) as the only Rh‐containing species. Complex **9** was identified by comparison of its spectroscopic data with those of an independently prepared pure sample, obtained from the reaction of [Rh(Tp)(PHPh_2_)(C_2_H_4_)] with fumCN (Figure S10).

Consistent with this observation, DFT‐calculated thermodynamics for the reactions: [Rh(*κ* ^2^‐Tp)(PHPh_2_)(PRPh_2_)] (**6**, **7**) + olefin → [Rh(Tp)(PHPh_2_)(olefin)] (**8**, **9**) + PRPh_2_ yield a Δ*G*_298_ of +1.6 kcal mol^−1^ for dimethyl fumarate, indicating a slightly unfavorable process. In contrast, the reaction with fumaronitrile is thermodynamically favorable (Δ*G*_298_ = −1.6 kcal mol^−^^1^). This difference significantly reduces the steady‐state concentration of complex **1** in the presence of fumCN, leading to a dramatic decrease in turnover frequency.

To probe this point, the stoichiometric reactions of [Rh(Tp)(PHPh_2_)(RCH = CHR)] (R = CO_2_Me, **8**; CN, **9**) with a slight excess of PHPh_2_ (2.3 equiv.) were analyzed. Complex **8** reacts immediately with PHPh_2_ to afford complex **1** together with the functionalized phosphane Ph_2_P−CH(CO_2_Me)CH_2_CO_2_Me. In contrast, complex **9** reacts much more slowly yielding Ph_2_P−CH(CN)CH_2_CN after approximately 11 h under similar conditions. In this case, a plot of [NC−CH═CH−CN] versus time displays a clear exponential decay, consistent with first‐order kinetics, most likely associated with olefin decoordination. The observed rate constant, *k*_obs_ = 8.6 10^−5^ M s^−1^, corresponds to a first‐order rate constant *k* = 3.58 10^−3^ s^−1^ ([CN−CH═CH−CN]_0_ = 0.024 M). Application of the Eyring–Polanyi equation [34] yields a free‐energy barrier of approximately 20.8 kcal mol^−1^ at 298 K, which accounts for the experimentally observed slowdown of the catalytic process.

A third example of electrophilic hydrophosphanation was provided by the non‐activated 3‐hexene, eventually giving [Rh(*κ* ^2^‐Tp)(PHPh)(PRPh_2_)] (**10**, R = CH(Et)CH_2_Et). As with fumaronitrile, only one transition state (**TSb′′**) was located, again characterized by an elongated Rh−H bond (1.907 Å) and a forming C−H bond (1.250 Å). However, **TSb′′** lies too high in energy (33.0 kcal mol^−^^1^, Figure S3) to allow catalysis under the standard conditions.

**SCHEME 4:**
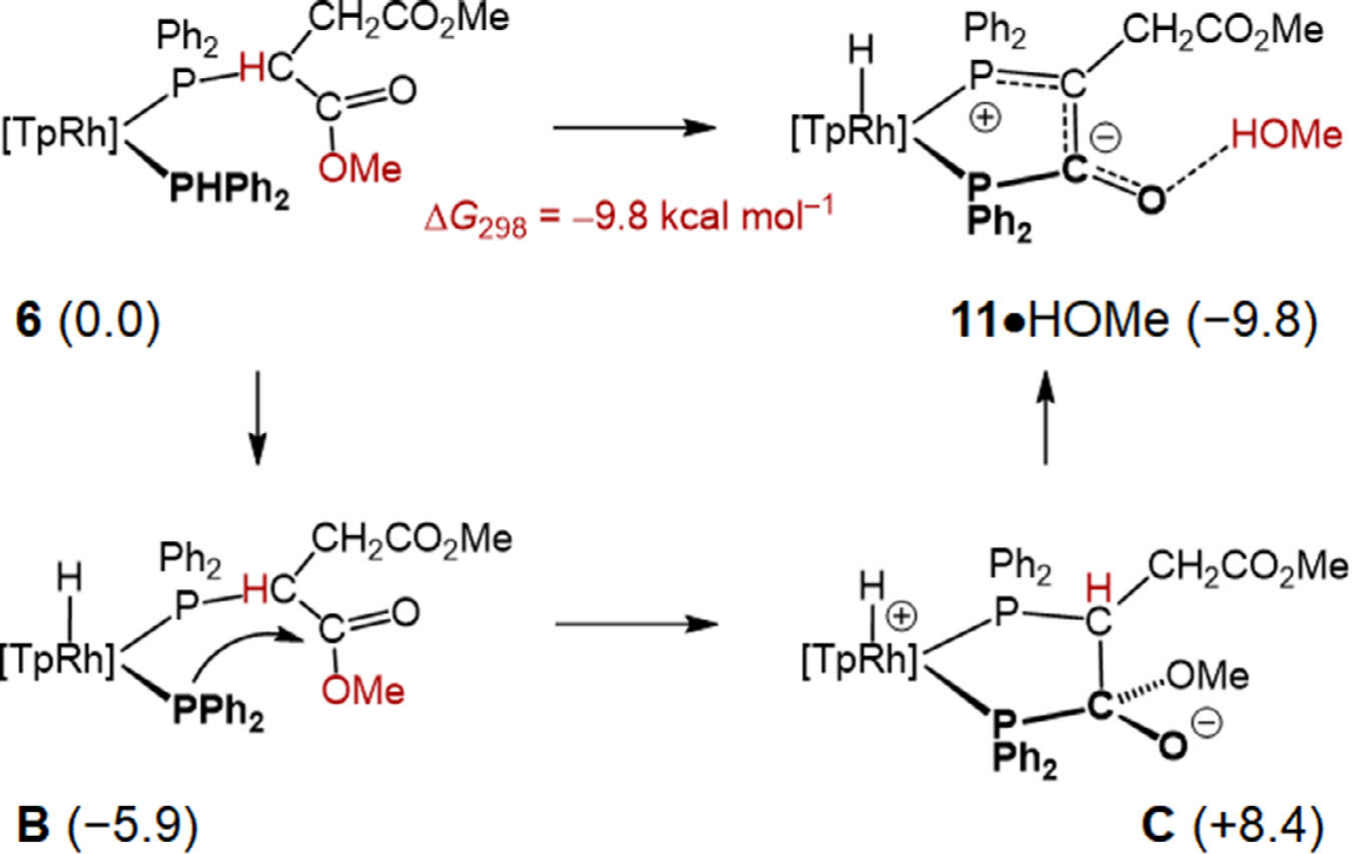
Elemental steps for the transformation of **6** into **11**. Relative Δ*G*_298_ values (in brackets) are given in kcal mol^−1^.

At the stoichiometric level, [Rh(Tp)(H)(PHPh_2_)(PPh_2_)] (**1**) reacts almost instantly with one mol‐equiv. of dimethyl fumarate to afford [Rh(κ^2^‐Tp)(PHPh_2_)(PRPh_2_)] (**6**, R = CH(CO_2_Me)CH_2_CO_2_Me) as the main product. Alternatively, complex **6** can also be straightforward prepared by reacting [Rh(Tp)(PHPh_2_)(C_2_H_4_)] with the corresponding phosphane PRPh_2_ (see the Supporting Information). Complex **6** is unstable in solution and slowly converts at room temperature into [Rh(Tp)(H)(Ph_2_PC(CO)CH_2_(CO_2_Me)─PPh_2_)] (**11**) over ∼ 24 h. This transformation occurs through a multistep process involving methanol extrusion, which was experimentally confirmed (Scheme 4). DFT calculations on the methanol adduct **11**•HOMe showed that it is 9.8 kcal mol^−1^ more stable than complex **6**, demonstrating that the conversion of **6** into **11**•HOMe is thermodynamically favorable.

Complex **11** was isolated as a white solid following standard work‐up procedures and fully characterized by elemental analysis and multinuclear NMR spectroscopy, as detailed in the Supporting Information. In solution, the hydride ligand and the single OMe group appear at *δ* = −14.29 and 3.43 ppm, respectively, in the ^1^H NMR spectrum. The two methylenic protons correlate with a carbon at δ = 34.3 ppm in the ^1^H,^13^C‐hsqc spectrum. In addition, the two phosphorus atoms, both coupled to rhodium (*J*(P,Rh) = 115 and 108 Hz), were observed as two doublets of doublets in the ^31^P{^1^H} NMR spectrum. Finally, the carbons involved in the enolate fragment (O−C═C) gave signals at *δ* = 179.6 and 86.6 ppm, respectively, in the ^13^C{^1^H} NMR spectrum.

**FIGURE 2:**
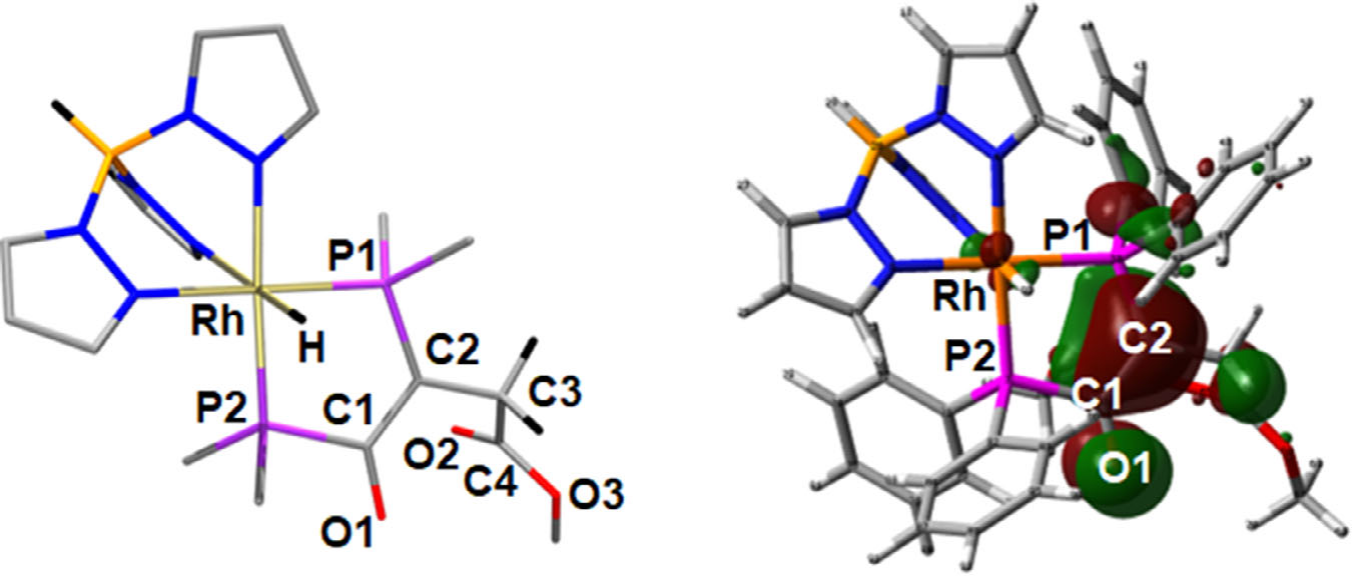
DFT‐calculated molecular structure (left) and HOMO (right) of complex **11**. Selected bond distances (Å) and angles (°): Rh−P1 2.317, Rh−P2 2.286, P1−C2 1.769, C1−C2 1.386, P2−C1 1.885, C1−O1 1.251, C2−C3 1.512, C4−O2 1.210, C4−O3 1.340. Code color: Rh (yellow), P (purple), O (red), N (blue), C (gray), B (orange), and H (black).

Figure 2 (left) shows the modeled structure of complex **11**. The O1─C1═C2 enolate fragment exhibits pronounced π‐delocalization, as indicated by intermediate bond lengths between typical single and double bonds. The shorter P1─C2 bond compared to the P2─C1 bond suggests P1 also contributes to the delocalization, which is consistent with the longer Rh─P1 bond relative to Rh─P2. This delocalization is further supported by the HOMO (Figure 2, right), which features a bonding interaction across the P1─C2─C1 unit and antibonding character with O1.

**SCHEME 5:**
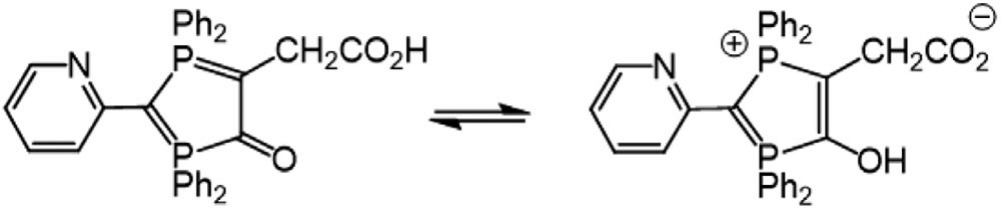
Tautomeric forms of the diphosphaenolate are shown in the Scheme.

A search in the Cambridge Structural Database (CSD) revealed that diphosphaenolates of the type “P(CO)CP” are virtually unknown. To date, only a single example: the pyridyl substituted derivative shown in Scheme 5 has been reported. In that case, the authors described the compound as existing in equilibrium between the two tautomeric forms depicted in the Scheme [35]. While such tautomerism is not feasible in our system, the structure of the pyridyl analogue lends strong support to the presence of significant π‐delocalization along the diphosphaenolate ring, as proposed for **11**.

A plausible reaction pathway for the conversion of complex **6** into complex **11** is depicted in Scheme 4. This multistep process is proposed to commence with the oxidative addition of the P−H bond, affording the hydrido‐phosphanido intermediate **B**. Notably, this species is calculated to be 5.9 kcal mol^−1^ more stable than the Rh(I) precursor **6**, underscoring the thermodynamic viability of the initial activation step. Importantly, this transformation enables a distinctive intramolecular reactivity, whereby the phosphanido ligand performs nucleophilic attack at the adjacent carbonyl carbon to generate intermediate **C**. Subsequent intramolecular proton abstraction then leads to the formation of complex **11**. Monitoring the reaction by NMR spectroscopy shows only complexes **6** and **11** to be present in solution, indicating that the rate‐determining step is the P─H bond activation reaction (**6** → **B**).

Complex **11** is inactive in the hydrophosphanation of dimethyl fumarate, resulting in partial catalyst deactivation at later stages of the reaction. To mitigate this effect, a slight excess of PHPh_2_ was employed under the catalytic conditions (**1**:PHPh_2_:fumarate = 1:110:100). This strategy takes advantage of favorable phosphane exchange, reducing the lifetime of complex **6** in solution

$$\begin{array}{ccc} 6 & + & \mathrm{PHP}h_{2}\to 1+Ph_{2}P-\mathrm{CH}\left(CO_{2}\mathrm{Me}\right)CH_{2}\left(CO_{2}\mathrm{Me}\right),\\ & & \Delta G=-1.5\;\mathrm{kcal}\mathrm{mo}l^{-1} \end{array}$$

Non‐symmetric olefins were also hydrophosphanated by complex **1** (Table 2). In the case of methyl acrylate, the reaction proceeds essentially instantaneously, even at low catalyst loading (1 mol%, highlighted in red). Plots of [olefin] *versus* time showed excellent exponential behavior, consistent with step 1 in Scheme 3 as the rate‐determining step (Figure S21). Moreover, a kinetic isotope effect (KIE) of 4.0 further supports the electrophilic mechanistic pathway proposed herein (Figure S20).

**TABLE 2:** Hydrophosphanation of non‐symmetric olefins catalyzed by complex **1**.^a^

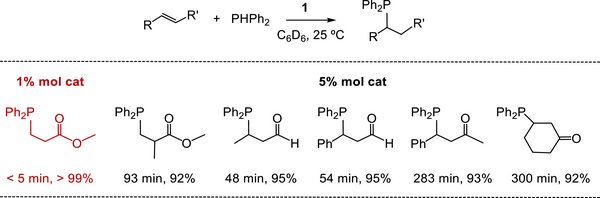

^a^ Reaction conditions: PHPh_2_ (0.48 mmol) and alkene (0.48 mmol), catalyst (0.024 mmol, 5% mol) in C_6_D_6_ (V_t_ = 0.7 mL) at 25°C. For catalytic loadings of 1%, the appropriate amount of catalyst was taken from a stock solution and the total volume was adjusted to 0.7 mL, [PHPh_2_] = [olefin] = 0.46 M (methyl acrilate). Conversions were determined by ^1^H NMR spectroscopy.

Other olefins, including methyl methacrylate and substrates bearing aldehyde (crotonaldehyde, cinnamaldehyde) or ketone (benzylideneacetone, cyclohexenone) functionalities, likewise afforded the corresponding anti‐Markovnikov phosphanes in high yields. However, higher catalyst loadings (5 mol%) were required to achieve full conversion within 1–5 h (Table 2, in black). This behavior is consistent with a change in the rate‐determining step to step 2 (phosphane exchange, Scheme 3). Accordingly, the data fit well to a straight line when plotting ${\left[PH\right]}^{0}Ln\left[PH\right]-\left[PH\right]$ $\left(PH=PHPh_{2}\right)$ versus time (Figure S22) [31].

## 3 Conclusions

In conclusion, we have uncovered a non‐classical, electrophilic hydride‐driven mechanism for the catalytic hydrophosphanation of olefins with PHPh_2_, marking a fundamental departure from traditional phosphanide nucleophilic pathways. DFT analysis and KIE measurements reveal this reactivity mode is general across both activated and non‐activated relevant olefins as well as for symmetric and non‐symmetric olefins. In parallel, we identified an unprecedented intramolecular transformation whereby a hydrido‐phosphanido complex undergoes olefin coupling to form a unique diphosphaenolate. This reactivity clearly reveals the dual electrophilic/nucleophilic character of the key complex **1**. Furthermore, we anticipate that these insights will stimulate the development of new catalytic systems, broaden the application of P─C bond‐forming strategies, and expand the conceptual framework of electrophilic hydride reactivity in synthesis and materials chemistry.

## Conflicts of Interest

The authors declare no conflicts of interest.

## Supporting information




**Supporting File**: The authors have cited additional references within the Supporting Information [36, 37, 38, 39, 40, 41].

## Data Availability

The data that support the findings of this study are openly available in digital csic at https://digital.csic.es/.
