# Supplementary material for: Breaking the Mold: Electrophilic Hydrophosphanation Emerges
Source: Chemistry. 2026 May 14;32(32):e71103. doi: 10.1002/chem.71103 (PMC13505727; doi:10.1002/chem.71103)
Supplement: Supplementary file 1 [file CHEM-32-e71103-s001.pdf]

# Breaking the Mold: Electrophilic Hydrophosphanation Emerges

Víctor Varela-Izquierdo, Janeth Navarro, Judit Cano-Asensio, José A. López, and Cristina Tejel*

Departamento de Química Inorgánica, Instituto de Síntesis Química y Catálisis Homogénea (ISQCH), CSIC-Universidad de Zaragoza, Pedro Cerbuna 12, 50009-Zaragoza (Spain)

## Table of contents

| | |
|---|---|
| Starting materials and physical methods | S1 |
| Synthesis and characterization of the complexes | S1 |
| Catalytic essays and synthesis of the phosphanes | S2 |
| Monitoring the reactions | S3 |
| Kinetic Isotope Effects | S4 |
| DFT-studies | S5 |
| NMR spectra | S7 |
| Catalytic kinetic profiles | S13 |
| References | S14 |

## Starting materials and physical methods.

All of the operations were carried out under an argon atmosphere using standard Schlenk techniques as well as dry-box facilities. Complexes [Rh(Tp)($PHPh_2$)(H)($PPh_2$)] (**1**), [Rh(Tp)($PMe_3$)(H)($PPh_2$)] (**2**), [Rh(Tp)($PMe_2Ph$)(H)($PPh_2$)] (**3**), [Rh(Tp)($PMePh_2$)(H)($PPh_2$)] (**4**), [Rh(Tp)(IMes)($PHPh_2$)] (**5**) and [Rh(Tp)($PHPh_2$)($C_2H_4$)] were prepared according to literature methods.[S1] Diphenylphosphane purchased from Aldrich was found to contain ca. 4% of diphenylphosphane oxide. The oxide was removed by silica gel column chromatography using diethyl ether as eluent. Diethyl ether was then evaporated under vacuum and *trans*-NC−CH=CH−CN (fumCN) was purified by sublimation at 60 ºC (atmospheric pressure). Carbon, hydrogen, and nitrogen analyses were carried out with a Perkin-Elmer 2400 CHNS/O microanalyzer. NMR spectra were recorded on Bruker AV300 and AV500 spectrometers operating at 300.13 and 500.13 MHz for $^1$H, respectively. Chemical shifts are reported in ppm and referenced to $SiMe_4$, using the internal signal of the deuterated solvent ($^1$H and $^{13}$C) and external $H_3PO_4$ 85% in water ($^{31}$P). The high-resolution mass spectrometry (HRMS) measurements were carried out using Bruker MicrOTOF-Q mass spectrometers equipped with an electrospray ionization (ESI) source and a hybrid Quadrupole–Time-of-Flight (Q-TOF) analyzer or a Bruker TIMS-TOF-FLEX mass spectrometer.

## Synthesis and characterization of the complexes.

**[Rh(Tp)($PHPh_2$)(fum)]** (**8**). Diphenylphosphane (50.0 µL, 0.287 mmol) was firstly added to a solution of [Rh(Tp)($C_2H_4$)$_2$] (106.7 mg, 0.287 mmol) in toluene (5 mL). After 10 minutes of reaction, ethylene was removed under reduced pressure and solid dimethyl fumarate (fum, 41.4 mg, 0.287 mmol) was added. After stirring for 30 min, the solvent was evaporated to yield the product as a yellow solid, which was washed with hexane (2 x 5 mL) and vacuum-dried. Yield: 150.6 mg (81.2 %) (Figure S5).

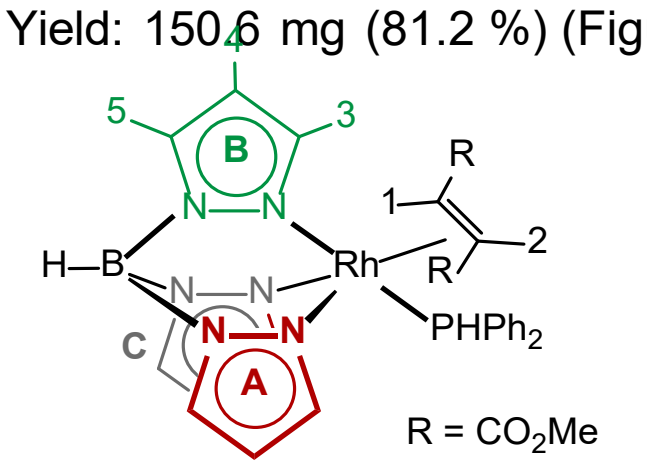


$^1$H NMR (400.13 MHz, $C_6D_6$, 25 ºC): δ = 8.69 (d, $^3J$(H,H) = 2.7 Hz, 1H, $Pz^{B3}$), 7.98 (ddt, $^3J$(H,H) = 11.2 Hz, $^3J$(H,P) = 5.9 Hz, $^4J$(H,H) =1.6 Hz, 2H, $PPh^{o1}$), 7.80 (d, $^3J$(H,H) = 1.8 Hz, 1H, $Pz^{A3}$), 7.56 (dd, $^3J$(H,H) = 2.4 Hz, $^5J$(H,P) = 0.7 Hz, 1H, $Pz^{A5}$), 7.42 (dd, $^3J$(H,H) = 2.4 Hz, $^5J$(H,P) = 0.7 Hz, 1H, $Pz^{C5}$), 7.30 (dt, $^3J$(H,H) = 2.3 z, $^4J$(H,H) = $^4J$(H,P) = 1.1 Hz, 1H, $Pz^{B5}$), 7.10 (d, $^3J$(H,H) = 2.0 Hz, 1H, $Pz^{C3}$), 7.06 (m, 3H, $PPh^{(m+p)1}$), 6.76 (tq, $^3J$(H,H) = 6.9 Hz, $^4J$(H,H) = $^5J$(H,P) = 1.6 Hz, 1H, $PPh^{p2}$), 6.65 (m, 2H, $PPh^{m2}$), 6.57 (m, 2H, $PPh^{o2}$), 6.15 (d, $J$(H,P) = 386.8 Hz, 1H, PH), 5.89 (t, $^3J$(H,H) = 2.1 Hz, 1H, $Pz^{A4}$), 5.79 (td, $^3J$(H,H) = 2.3 Hz, $^5J$(H,P) = 1.2 Hz, 1H, $Pz^{B4}$), 5.60 (t, $^3J$(H,H) = 2.1 Hz, 1H, $Pz^{C4}$), 4.62 (dd, $^3J$(H,H) = 9.7 Hz, $^2J$(H,Rh) = 2.1 Hz, 1H, $H^1$), 4.08 (ddd, $^3J$(H,H) = 9.7 Hz, $^3J$(H,P) = 7.6, $^2J$(H,Rh) = 2.0 Hz, 1H, $H^2$), 3.55 (s, 3H) and 3.20 (s, 3H) (OMe).

$^{31}$P{$^1$H} NMR (162.0 MHz, $C_6D_6$, 25 ºC): δ = 36.4 (d, $J$(P,Rh) = 138 Hz, $PHPh_2$).

IR (ATR): ν(B-H)/cm$^{-1}$ 2473 (m), ν($CO_2Me$)/cm$^{-1}$ 1692 (sh), 1682 (s).

HRMS (ESI$^+$): *m/z* (%):calcd for $C_{27}H_{29}N_6BNaO_4PRh$ [*M*+Na]$^+$ 669.1032, found 669.1033 (err [mDa] = 0.1).

Anal. calcd (%) for $C_{27}H_{29}N_6BO_4PRh$ (646.25): C 50.18, H 4.52, N 13.00; found: C 51.29, H 4.60, N 12.99.

**[Rh(Tp)($PHPh_2$)(fumCN)]** (**9**) was prepared as above starting from [Rh(Tp)($C_2H_4$)$_2$] (37.2 mg, 0.10 mmol), diphenylphosphane (17.4 µL, 0.10 mmol) and fumaronitrile (fumCN, 7.8 mg, 0.10 mmol). Yield: 50.3 mg (86.7 %) (Figure S6).

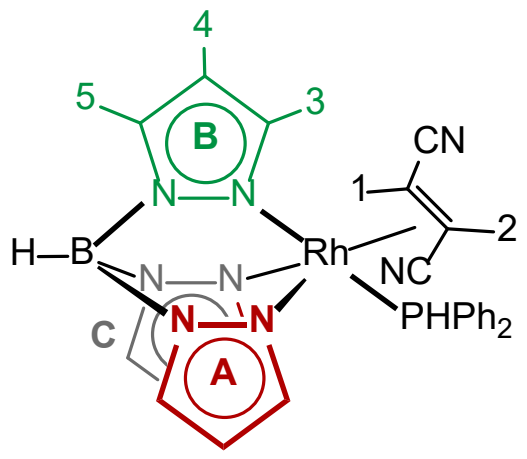

$^1$H{$^{31}$P} NMR (500.13 MHz, $C_6D_6$, 25 ºC): δ = 7.66 (d, $^3J$(H,H) = 2.1 Hz, 1H, $Pz^{B3}$), 7.58 (d, $^3J$(H,H) = 7.5 Hz, 2H, $Ph^{o1}$), 7.51 (d, $^3J$(H,H) = 2.3 Hz, 1H, $Pz^{A3}$), 7.48 (d, $^3J$(H,H) = 1.9 Hz, 1H, $Pz^{A5}$), 7.38 (d, $^3J$(H,H) = 2.3 Hz, 1H, $Pz^{C5}$), 7.28 (d, $^3J$(H,H) = 1.9 Hz, 1H, $Pz^{C3}$), 7.26 (d, $^3J$(H,H) = 1.9 Hz, 1H, $Pz^{B5}$), 6.98 (t, $^3J$(H,H) = 7.1 Hz, 1H, $Ph^{p1}$), 6.92 (t, $^3J$(H,H) = 7.4 Hz, 2H, $Ph^{m1}$), 6.70 (t, $^3J$(H,H) = 7.5 Hz, 1H, $Ph^{p2}$), 6.55 (t, $^3J$(H,H) = 7.6 Hz, 2H, $Ph^{m2}$), 6.40 (d, $^3J$(H,H) = 7.7 Hz, 2H, $Ph^{o2}$), 6.24 (d, $J$(HP) = 378.0 Hz, 1H), 5.75 (d, $^3J$(H,H) = 2.1 Hz, 1H, $Pz^{A4}$), 5.74 (d, $^3J$(H,H) = 2.1 Hz, 1H, $Pz^{B4}$), 5.63 (d, $^3J$(H,H) = 2.1 Hz, 1H, $Pz^{C4}$), 2.86 (dd, $^3J$(H,H) = 8.8 Hz, $^2J$(H,Rh) = 2.3 Hz, 1H, $H^1$), 2.43 (dd, $^3J$(H,H) = 8.7 Hz, $^2J$(H,Rh) = 1.9 Hz, 1H, $H^2$).

$^{31}$P{$^1$H} NMR (202.46 MHz, $C_6D_6$, 25 ºC): 35.0 (d, $J$(P,Rh) = 133 Hz).

IR (ATR): ν(B-H)/cm$^{-1}$ 2481 (m), ν(CN)/cm$^{-1}$ 2206 (s).

HRMS (TIMS+): *m/z* (%): calcd for $C_{25}H_{23}N_8BNaPRh$ [*M*+Na]$^+$ 603.0828, found 603.0900 (err [mDa] = 7.2); for $C_{25}H_{23}N_8BPRhH$ [*M*+H]$^+$ 581.1009, found 581.1076 (err [mDa] = 6.7). HRMS (TIMS-): *m/z* (%): calcd for $C_{25}H_{22}N_8BPRh$ [*M*-H]$^-$ 579.0853, found 579.0801 (err [mDa] = 7.2).

Anal. calcd (%) for $C_{25}H_{23}N_8BPRh$ (580.1940): C 51.75, H 4.00, N 19.31; found: C 51.67, H 3.75, N 18.31.

**[Rh(Tp)(H)($Ph_2PC(CO)CH_2(CO_2Me)$–$PPh_2$)]** (**11**). A solution of $PHPh_2$ (49.1 μL, 0.282) in toluene (3 mL) was added dropwise to a yellow solution of [Rh(Tp)$(C_2H_4)_2$] (105.1 mg, 0.282 mmol) in toluene (5 mL). After 10 minutes of reaction, ethylene was removed under reduced pressure. Subsequently, solid $Ph_2P$−$CH(CO_2Me)CH_2CO_2Me$ (22, 98.0 mg, 0.297 mmol) was added, and the mixture was allowed to react for 48 h. The resulting colorless solution was concentrated to *ca* 1 mL, and hexane (5 mL) was added. The white solid that precipitated was decanted, washed in hexane (2 x 2 mL) and dried under vacuum. Yield: 178.1 mg (79%) (Figures S7, S8).

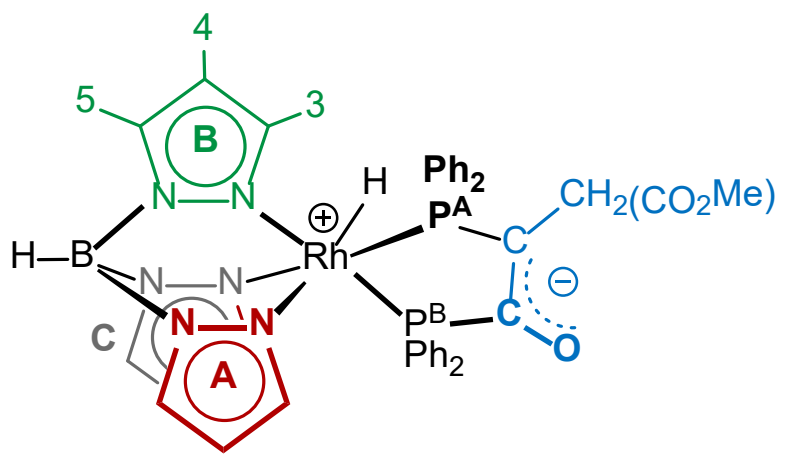


$^1$H NMR (500.13 MHz, $C_6D_6$, 25 °C): δ = 8.10 (ddd, $^3J$(H,H) = 11.8 Hz, $^3J$(H,P) = 8.2 Hz, $^4J$(H,H) = 1.3 Hz, 2H, $PPh^{Bo1}$), 8.01 (ddd, $^3J$(H,H) = 12.1 Hz, $^3J$(H,P) = 8.0 Hz, $^4J$(H,H) = 1.6 Hz, 2H, $PPh^{Ao1}$), 7.85 (ddd, $^3J$(H,H) = 10.0 Hz, $^3J$(H,P) = 8.4 Hz, $^4J$(H,H) = 1.5 Hz, 2H, $PPh^{Bo2}$), 7.40 (ddd, $^3J$(H,H) = 11.4 Hz, $^3J$(H,P) = 8.2 Hz, $^4J$(H,H) = 1.5 Hz, 2H, $PPh^{Ao2}$), 7.33 (dt, $^3J$(H,H) = 2.3 Hz, $^4J$(H,H) = $^5J$(H,P) = 0.8 Hz, 1H, $Pz^{B5}$), 7.30 (dt, $^3J$(H,H) = 2.4 Hz, $^4J$(H,H) = $^5J$(H,P) = 0.9 Hz, 1H, $Pz^{C5}$), 7.24 (dt, $^3J$(H,H) = 2.4 Hz, $^4J$(H,H) = $^5J$(H,P) = 0.6 Hz, 1H, $Pz^{A5}$), 7.16 (overlapped with $C_6D_6$, 1H, $Pz^{C3}$), 7.15 (td, $^3J$(H,H) = 7.4 Hz, $^4J$(H,H) = 1.4 Hz, 2H, $PPh^{Bm1}$), 7.07 (m, 4H, $PPh^{Bp1}$+$Ph^{A(m+p)1}$), 6.90 (d, $^3J$(H,H) = 1.9 Hz,1H, $Pz^{B3}$), 6.87 (m, 1H, $PPh^{Bp2}$), 6.83 (tdd, $^3J$(H,H) = 6.9 Hz, $^4J$(H,P) = 2.4 Hz, $^4J$(H,H) = 0.9 Hz, 2H ,$PPh^{Bm2}$), 6.80 (m, 1H, $PPh^{Ap2}$), 6.77 (m, 2H, $PPh^{Am2}$), 5.94 (d, $^3J$(H,H) = 2.3 Hz, 1H, $Pz^{A3}$), 5.62 (td, $^3J$(H,H) = 2.2 Hz, $^5J$(H,P) = 0.7 Hz, 1H, $Pz^{B4}$), 5.60 (td, $^3J$(H,H) = 2.2 Hz, $^5J$(H,P) = 1.0 Hz, 1H, $Pz^{C4}$), 5.30 (t, $^3J$(H,H) = 2, $^5J$(H,P) = 2 Hz, 1H, $Pz^{A4}$), 4.46 (br s, 1H, BH), 3.64 (ddd, $^2J$(H,H) = 16.4 Hz, $^3J$(H,P) = 11.8 Hz, $^4J$(H,P) = 1.1 Hz, 1H, $CH_2$), 3.43 (s, 3H, OMe), 3.35 (ddd, $^2J$(H,H) = 16.4, $^3J$(H,P) = 7.1 Hz, $^4J$(H,P) = 1.5 Hz, 1H, $CH_2$), −14.29 (ddd, $^2J$(H,P) = 18.1 and 16.7 Hz, *J*(H,Rh) = 13.5 Hz, 1H, Rh−H).

$^{31}$P{$^1$H} NMR (202.46 MHz, $C_6D_6$, 25 °C): δ = 58.2 (dd, *J*(P,Rh) = 115 Hz, $^2J$(P,P) = 40 Hz, 1P, $P^B$), 58.2 (dd, *J*(P,Rh) = 108 Hz, $^2J$(P,P) = 40 Hz, 1P, $P^A$).

$^{13}$C{$^1$H} NMR (128.8 MHz, $C_6D_6$, 25 °C): selected resonances, δ = 179.6 (ddd, *J*(C,P) = 47 Hz, $^2J$(C,P) = 44 Hz, $^2J$(C,Rh) = 4 Hz, C−*C*O), 173.0 (dd, $^3J$(C,P) = 5.1, $^4J$(C,P) = 1.5 Hz, $CO_2CH_3$), 86.6 (dd, *J*(C,P) = 98 Hz, $^2J$(C,P) = 95 Hz, $^2J$(C,Rh) = 1 Hz, *C*−CO), 51.2 (OMe), 34.3 (dd, $^2J$(C,P) = 15 Hz, $^3J$(C,P) = 5 Hz, $CH_2$).

IR (ATR): ν(B-H)/cm$^{-1}$ 2494 (m), ν($CO_2Me$)/cm$^{-1}$ 1736(s), 1722(s), ν(C-O)/cm$^{-1}$ 1552(m).

HRMS (ESI+): m/z, %: calc. for $C_{38}H_{36}N_6BO_3P_2Rh$ [*M*+H]$^+$ 801.1552, found: 801.1560 (error (mD) = −0.8).

Anal. Calc. (%) for $C_{38}H_{36}N_6BO_3P_2Rh$ (800.40): C 57.02, H 4.53, N 10.50; found: C 57.29, H 4.32, N 10.26.

## Catalytic essays and synthesis of the phosphanes

In a glovebox, the catalyst (0.024 mmol, 5%, complexes **1**-**5**) was accurately weighed into an NMR tube and dissolved in the 300 μL of $C_6D_6$. Then, the internal standard (dioxane, 5 μL), and a solution of $PHPh_2$ (83.1 μL, 0.48 mmol) and the olefin (0.48 mmol) in 400 μL of $C_6D_6$ were added (final volume = 0.7 mL). At this point, the chronometer was switched on. The NMR tube was sealed, removed from the glovebox and immediately loaded into the NMR spectrometer (around 3-5 min). $^1$H NMR spectra were recorded at different intervals of time, intercalating some $^{31}$P{$^1$H} NMR spectra to follow the fate of the catalyst. For reactions with catalyst **1**, a slight excess of $PHPh_2$ (86.6 μL, 0.50 mmol) was used to slow down further transformations of the diphosphane–rhodium(I) complexes. [Rh($\kappa^2$-Tp)($PHPh_2$)($PRPh_2$)] (R = $CH(CO_2Me)CH_2CO_2Me$, **6**; $CH(CN)CH_2CN$, **7**). Under these conditions, the $^{31}$P{$^1$H} NMR spectrum at the end of the catalytic reaction with *trans*-$MeO_2C$−CH=CH−$CO_2Me$ (fum) showed the clean formation of the phosphane $Ph_2P$−$CH(CO_2Me)CH_2CO_2Me$, while rhodium remained as complex **1** (Figure S9). On the contrary, the similar catalytic reaction with *trans*-NC−CH=CH−CN slows down significantly after the first catalytic cycle, although it completes in around one week (Figure S10). From these catalytic solutions the functionalized phosphanes were isolated as follows:

**$Ph_2P$–$CH(CO_2Me)CH_2CO_2Me$.** The catalytic solution was evaporated to dryness and the residue was chromatographed (silica) in the dry-box, using diethyl ether as eluent. Evaporation of the solvent renders $Ph_2P$−$CH(CO_2Me)CH_2CO_2Me$ as a white solid (98% yield) (Figures S11, S12).

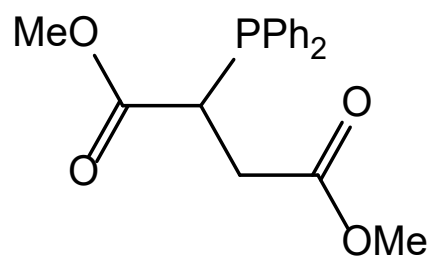


$^1$H NMR (300.13 MHz, $C_6D_6$, 25 ºC) δ = 7.55 (m, 2H, PPh$^{o1}$), 7.48 (m, 2H, PPh$^{o2}$), 7.03 (m, 6H, PPh$^{(m+p)1}$ + PPh$^{(m+p)2}$), 4.00 (dd, $^3J$(H,H) = 11.7, 3.7 Hz, $^2J$(H,P) = 1.2 Hz 1H, CH), 3.21 (s, 3H, $CH_2CO_2$*Me*), 3.18 (ddd, $^2J$(H,H) = 17.2 Hz, $^3J$(H,H) = 11.7 Hz, $^3J$(H,P) = 6.2 Hz, 1H) and 2.51 (ddd, $^2J$(H,H) = 17.2 Hz, $^3J$(H,H) = 3.7 Hz, $^3J$(H,P) = 6.8 Hz, 1H) ($CH_2$), 3.12 (s, 3H, $CHCO_2$*Me*).

$^{31}$P{$^1$H} NMR (121.5 MHz, $C_6D_6$, 25 ºC): δ = –0.15 (s).

$^{13}$C{$^1$H} NMR (75.5 MHz, $C_6D_6$, 25ºC): δ = 172.2 (d, $^3J$(C,P) = 4 Hz, $CH_2$*C*=O), 172.0 (d, $^2J$(C,P) = 15 Hz, CH*C*=O), 135.7 (d, *J*(C,P) = 17 Hz, PPh$^{i1}$), 135.5 (d, *J*(C,P) = 17 Hz, PPh$^{i2}$), 134.1 (d, $^2J$(C,P) = 21 Hz, PPh$^{o1}$), 133.8 (d, $^2J$(C,P) = 21 Hz, PPh$^{o2}$), 129.5 (d, $^4J$(C,P) = 7 Hz, PPh$^{p(1+2)}$), 128.9 (d, $^3J$(C,P) = 19 Hz, PPh$^{m1}$), 128.7 (d, $^3J$(C,P) = 20 Hz, PPh$^{m2}$), 51.4 ($CH_2CO_2$*Me*), 51.37 (2C, $CHCO_2$*Me*), 40.6 (d, *J*(C,P) = 25 Hz, CH), 34.8 (d, $^2J$(C,P) = 22 Hz, $CH_2$).

HRMS (m/z): calcd for $C_{18}H_{19}NaO_4P$ [*M*+Na]$^+$ 353.0913, found 353.0920 (err [mDa] = –0.7).

**$Ph_2P$–$CH(CN)CH_2CN$**. The catalytic solution was evaporated to dryness and the residue was washed with hexane (2 x 1 mL) to remove the excess of $PHPh_2$. Then, the residue was extracted with diethyl ether (2 x 1 mL) and filtered. After that, hexane (10 mL) was added to the pale-yellow filtrate and left overnight. The suspension was filtered again and evaporation of the solvent to dryness gives $Ph_2P$−$CH(CN)CH_2CN$ as a pale-yellow oil (Figures S13, S14).

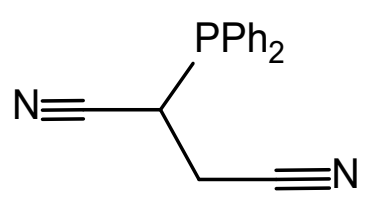


$^1$H NMR (300.13 MHz, $C_6D_6$, 25 ºC) δ = 7.44 (m, 2H, PPh$^{o1}$), 7.12 (m, 2H, PPh$^{o2}$), 7.02 (m, 6H, PPh$^{(m+p)1}$ + PPh$^{(m+p)2}$), 2.54 (m, 1H, CH), 1.62 (t, $^3J$(H,H) = 7.8 Hz, 2H, $CH_2$).

$^{31}$P{$^1$H} NMR (161.98 MHz, $C_6D_6$, 25 ºC): δ = –4.11 (s).

$^{13}$C{$^1$H} NMR (100.62 MHz, $C_6D_6$, 25ºC): δ = 134.7 (d, $^2J$(C,P) = 22 Hz, PPh$^{o1}$), 133.4 (d, *J*(C,P) = 14 Hz, PPh$^{i1}$), 132.6 (d, $^2J$(C,P) = 19 Hz, PPh$^{o2}$), 132.1 (d, *J*(C,P) = 17 Hz, PPh$^{i2}$), 131.1 (s, PPh$^{p1}$), 130.0 (s, PPh$^{p2}$), 129.22 (d, $^3J$(C,P) = 8 Hz, PPh$^{m1}$), 129.19 (d, $^3J$(C,P) = 6 Hz, PPh$^{m2}$), 117.1 (d, $^3J$(C,P) = 1 Hz, N*C*$CH_2$), 115.8 (d, $^2J$(C,P) = 11 Hz, N*C*CH), 26.1 (d, *J*(C,P) = 32 Hz, CH), 18.3 (d, $^2J$(C,P) = 25 Hz, $CH_2$).

HRMS (ESI$^+$): *m/z* (%): calcd for $C_{16}H_{14}N_2P$ [*M*+H]$^+$ 265.0889, found 265.0889 (err [mDa] = 0).

## Monitoring the reactions.

**Reaction of [Rh(Tp)($PHPh_2$)($C_2H_4$)] with $Ph_2P$–$CH(CO_2Me)CH_2CO_2Me$**. Toluene-$d_8$ (0.5 mL) was added to an NMR tube containing solid [Rh(Tp)($PHPh_2$)($C_2H_4$)] (12.4 mg, 0.0233 mmol) and the phosphane $Ph_2P$−$CH(CO_2Me)CH_2CO_2Me$ (7.7 mg, 0.0233 mmol). A bright orange solution was immediately formed and two vacuum/argon cycles were performed to remove ethylene. The resulting reaction mixture was then analyzed by VT-NMR spectroscopy (Figure S15). Under these conditions, the spectra show complex [Rh($\kappa^2$-Tp)(PHPh)($PRPh_2$)] (**6**, R = $CH(CO_2Me)CH_2CO_2Me$) as three isomeric species along with minor amounts of [Rh(Tp)(H)($Ph_2PC(CO)CH_2(CO_2Me)$–$PPh_2$)] (**11**).

These fluxional species have been tentatively assigned to that shown in the Scheme (R = $CH(CO_2Me)CH_2CO_2Me$):

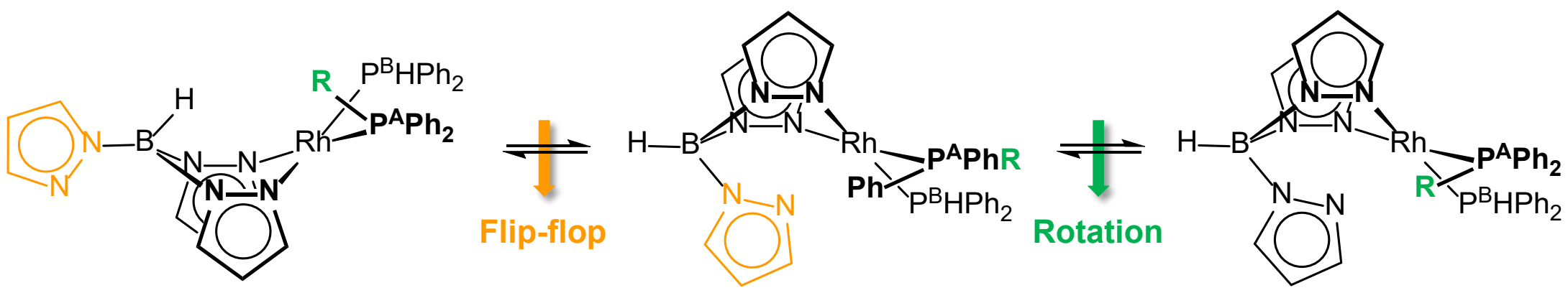


Two of them could be related by an inversion of the six-membered metallacycle 'B−(N−N)$_2$−Rh' (flip-flop) while the third one could correspond to the same rotational conformer arising from the rotation around the Rh−P bond as that shown in the Scheme.

The best-resolved spectrum was obtained at −10 °C; therefore, the sample was studied at this temperature. The three fluxional species **A**, **B**, and **C**, each display two inequivalent phosphorus atoms, as observed in the $^{31}$P,$^{31}$P{$^1$H}-cosy spectrum (Figure S16, down, left). The signals in the δ = 22–32 ppm region correspond to a phosphane $PHPh_2$ ligand, as

confirmed by the $^1$H,$^{31}$P-hmbc spectrum (Figure S16, down, right). The separation between the two cross-peaks is 352, 340, and 344 Hz for species **A**, **B**, and **C**, respectively. These values for the $J$(H,P) couplings are characteristic of protons directly bonded to phosphorous atoms. Accordingly, these PH protons resonate in the $^1$H NMR spectrum as doublet of doublets due to an additional smaller coupling $^2J$(H,P) ≈ 57 Hz, with the second phosphorous atom of the $PRPh_2$ ligand (Figure S17). Furthermore, the $J$($P^A$,Rh) values for **A**, **B**, and **C** (171, 166, and 162 Hz, respectively) are larger than those observed in BPT-complexes such as [Rh(Tp)($PHPh_2$)(olefin)] ($J$(P,Rh) = 138 Hz (fum), 133 Hz (fumCN)), thus indicating SP-geometries for species **A**-**C**. Unfortunately, the $CH(CO_2Me)CH_2CO_2Me$ fragment could be only identified for the major species **A** (Figure 17).

Selected spectroscopic data for **[Rh($\kappa^2$-Tp)(PHPh)($PRPh_2$)]** (**6**, R = $CH(CO_2Me)CH_2CO_2Me$)

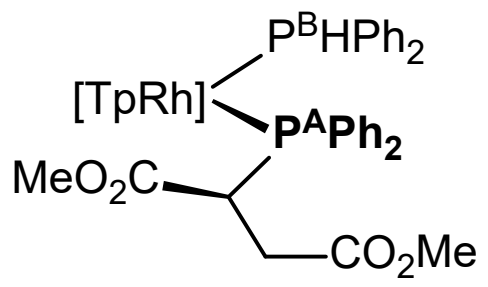


$^1$H NMR (400.13, toluene-$d_8$, −10 °C): **A**: $\delta$ = 5.49 (dd, $J$(H,P) = 352.0 Hz, $^3J$(H,P) = 16.4 Hz, 1H, PH), 4.39 (m 1H) and 4.27 (m, 1H) ($CH_2$), 4.16 (m, CH), 3.21 (s, 3H) and 3.09 (s, 3H) (OMe).

$^{31}$P{$^1$H} NMR (121.5 MHz, toluene-$d_8$, −10 ºC): **A**: $\delta$ = 62.4 (dd, $J$(P,Rh) = 174 Hz, $^2J$(P,P) = 56 Hz, $P^A$), 29.32 (dd, $^2J$(P,P) = 170 Hz, $^2J$(P,P) = 56 Hz, $P^B$); **B**: $\delta$ = 63.1 (dd, $J$(P,Rh) = 174 Hz, $^2J$(P,P) = 58 Hz, $P^A$), 24.68 (dd, $J$(P,Rh) = 167 Hz, $^2J$(P,P) = 58 Hz, $P^B$); **C**: 58.8 (broad dd, $P^A$), 23.9 (broad dd, $P^B$).

Species **A**, **B**, and **C** cleanly evolve to complex [Rh(Tp)(H)($Ph_2PC(CO)CH_2(CO_2Me)$–$PPh_2$)] (**11**) over a period of 24h at 25 ºC (Figure S 18).

**Reaction of [Rh(Tp)($PHPh_2$)(fum)] (8) with $PHPh_2$.** $PHPh_2$ (6.2 µL, 0.026 mmol) was added to a solution of [Rh(Tp)($PHPh_2$)(fum)] (**8**, 10.0 mg, 0.015 mmol). The NMR tube was sealed, removed from the glovebox and immediately loaded into the NMR spectrometer (around 3-5 min). $^1$H and $^{31}$P{$^1$H} NMR showed the full transformation of **8** into complex **1** and the phosphane $Ph_2P$–$CH(CO_2Me)CH_2CO_2Me$.

**Reaction of [Rh(Tp)($PHPh_2$)(fumCN)] (9) with $PHPh_2$.** An NMR tube was prepared as above starting from [Rh(Tp)($PHPh_2$)(fumCN)] (**9**, 7.0 mg, 0.012 mmol) and $PHPh_2$ (4.8 µL, 0.028 mmol) in $C_6D_6$ (0.5 mL). At this point, the chronometer was switched on. $^1$H NMR spectra were recorded at different intervals of time, intercalating some $^{31}$P{$^1$H} NMR spectra to follow the fate of the catalyst. Acquisition times were obtained directly from the MestReNova software (view/table/parameters). The kinetics were monitored by integration of the methylene resonance of $Ph_2P$ –$CH(CO_2Me)CH_2(CO_2Me)$ using the methyl signal of toluene as internal reference, and concentrations were calculated standard up to a conversion of ca 97%. A plot of [NC−CH=CH−CN] *versus* time displays a clear exponential decay, consistent with first-order kinetics, most likely associated with olefin decoordination. The observed rate constant, $k_{obs}$ = 8.6 $10^{-5}$ M $s^{-1}$, corresponds to a first-order rate constant $k$ = 3.58 $10^{-3}$ $s^{-1}$ ($[CN−CH=CH−CN]_0$ = 0.024 M) (Figure S19).

## Kinetic Isotope Effects

NMR tubes for kinetic isotope effect (KIE) measurements (intermolecular competition) were prepared by adding the corresponding olefin (0.246 mmol) to an NMR tube containing complex **1** (7.1 mg, 0.012 mmol) in $C_6D_6$ (0.27 mL). An equimolecular mixture of $Ph_2PH$:$Ph_2PD$ (1.23 mmol of each) was then added. When the catalysis reached completion the relative amount of deuterated to non-deuterated functionalized phosphane was measured by integration of quantitative $^1$H NMR spectra (Figure S20).

## DFT-studies

The DFT geometry optimizations were carried out with the Gaussian 09 program package,[S2] using the B3LYP-D3 dispersion-corrected hybrid functional.[S3–S6] Geometry optimizations were performed in the gas phase with the LanL2TZ(f) effective core potential basis set for the metal atoms and the 6-311G(d,p) basis set for the remaining ones. The LanL2TZ(f) basis set is based in the Hay-Wadt relativistic effective core potentials (RECPs) for the transition metal atoms.[S7] All minima (no imaginary frequencies) and transition states (one imaginary frequency) were characterized by calculating the Hessian matrix. The transition state search was performed with a relaxed PES scan of the key geometrical parameter and then the highest energy structure was optimized as a transition state by the default Gaussian 09 algorithms. The chemical correctness of the transition states found were confirmed by visual inspection of the normal mode having a negative vibrational frequency, followed by moving the TS geometries along the reaction path using the GaussView program utilities and reoptimizing to verify the nature of the products.

Cartesian coordinates for optimized (DFT) structures of complexes, intermediates, and transition states have been included in an independent xyz file.

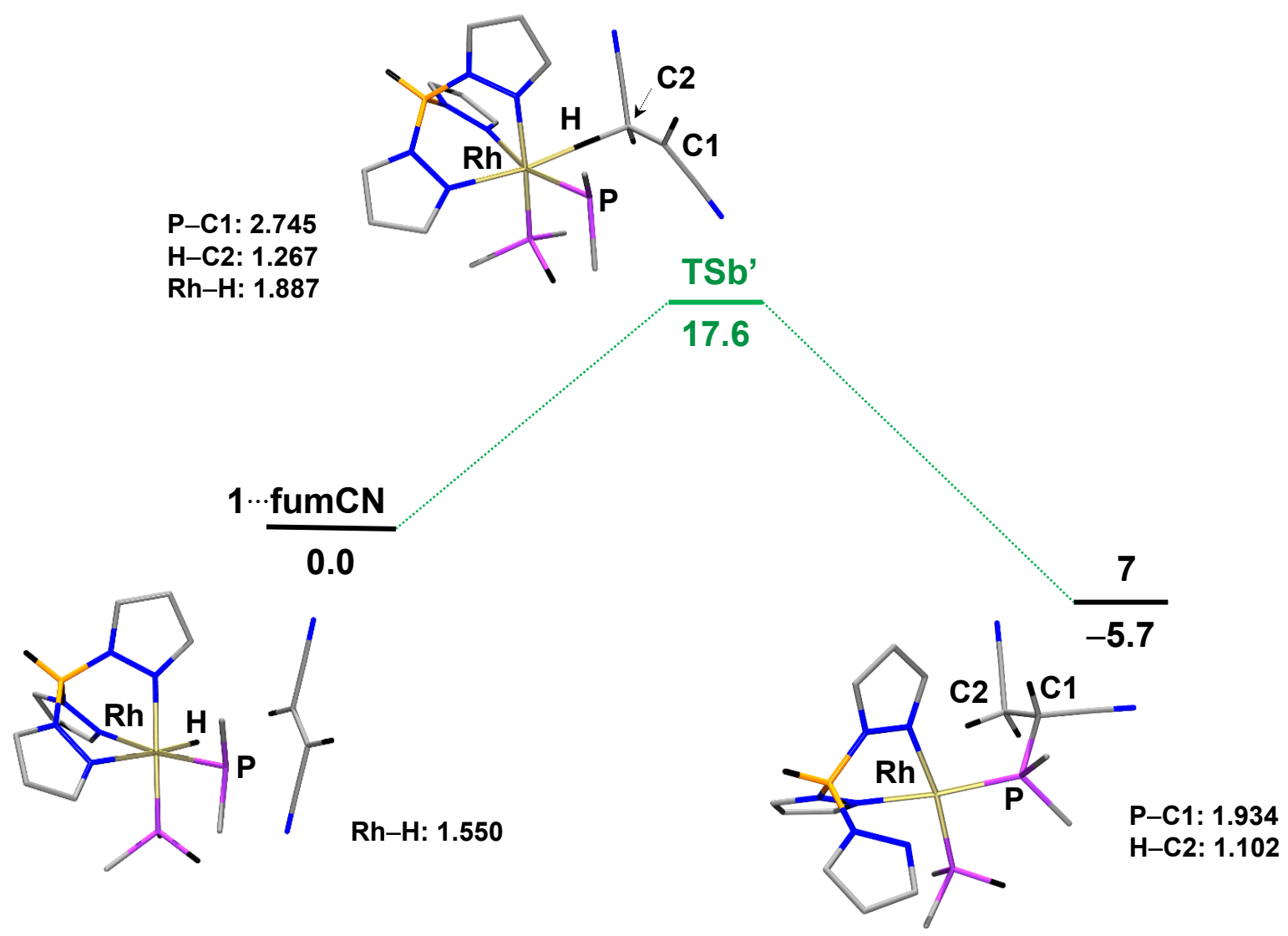


**Figure S1**. DFT computed Gibbs energy profile for the reaction of [Rh(Tp)(PHPh2)(H)(PPh2)] (**1**) with fumaronitrile. Relative $\Delta G_{298}$ values and selected bond distances are given in kcal mol$^{-1}$ and Å, respectively. Only the C$^{ipso}$ of the phenyl groups is shown for clarity. Color code: Rh (yellow), O (red), N (blue), C (gray), B (orange), H (black).

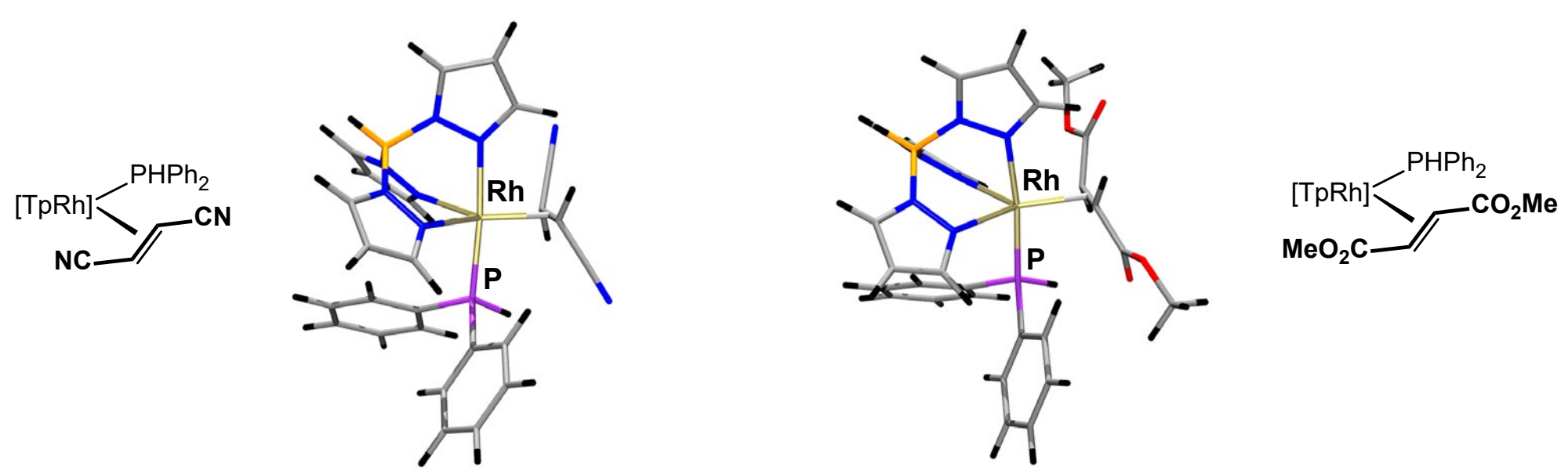


**Figure S2**. DFT modeled structures of [Rh(Tp)($PHPh_2$)(fumCN)] (**9**, left) and [Rh(Tp)($PHPh_2$)(fum)] (**8**, right). Color code: Rh (yellow), O (red), N (blue), C (gray), B (orange), H (black).

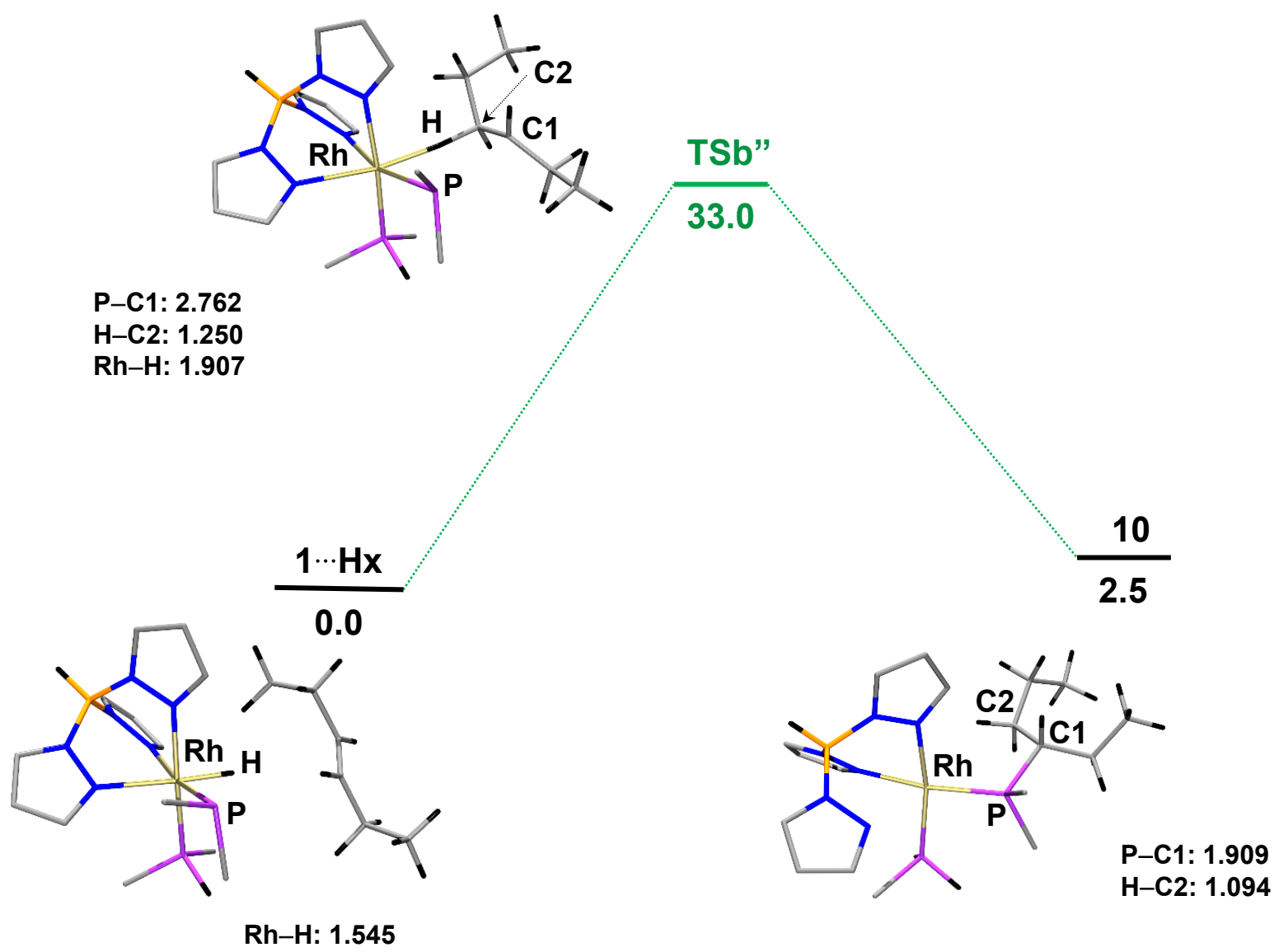


**Figure S3**. DFT computed Gibbs energy profile for the reaction of [Rh(Tp)(PHPh2)(H)(PPh2)] (**1**) with 3-hexene. Relative Δ$G_{298}$ values and selected bond distances are given in kcal mol$^{-1}$ and Å, respectively. Only the $C^{ipso}$ of the phenyl groups is shown for clarity. Color code: Rh (yellow), O (red), N (blue), C (gray), B (orange), H (black).

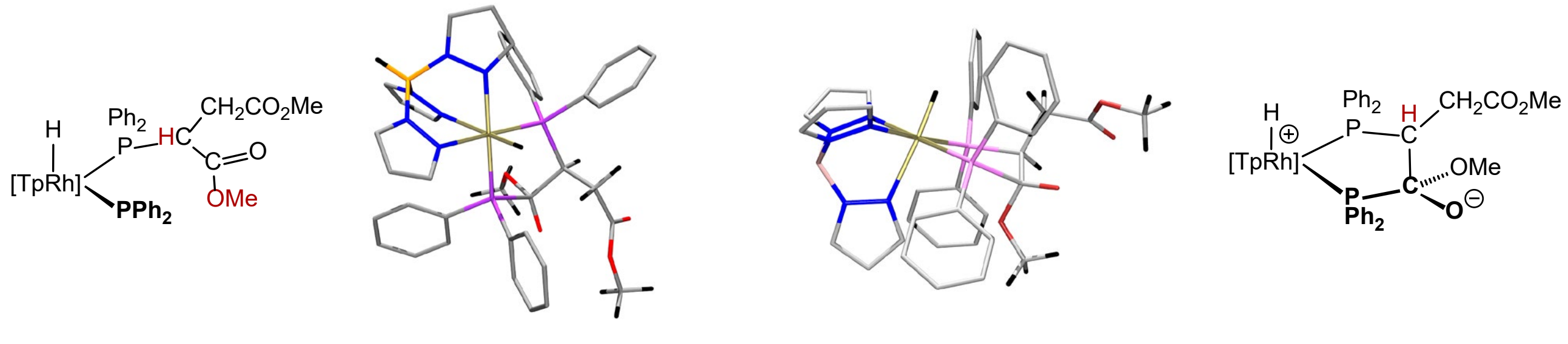


**Figure S4**. DFT modeled structures of species **B** (left) and **C** (right) in Scheme 4 (main text). Color code: Rh (yellow), O (red), N (blue), C (gray), B (orange), H (black).

## NMR spectra

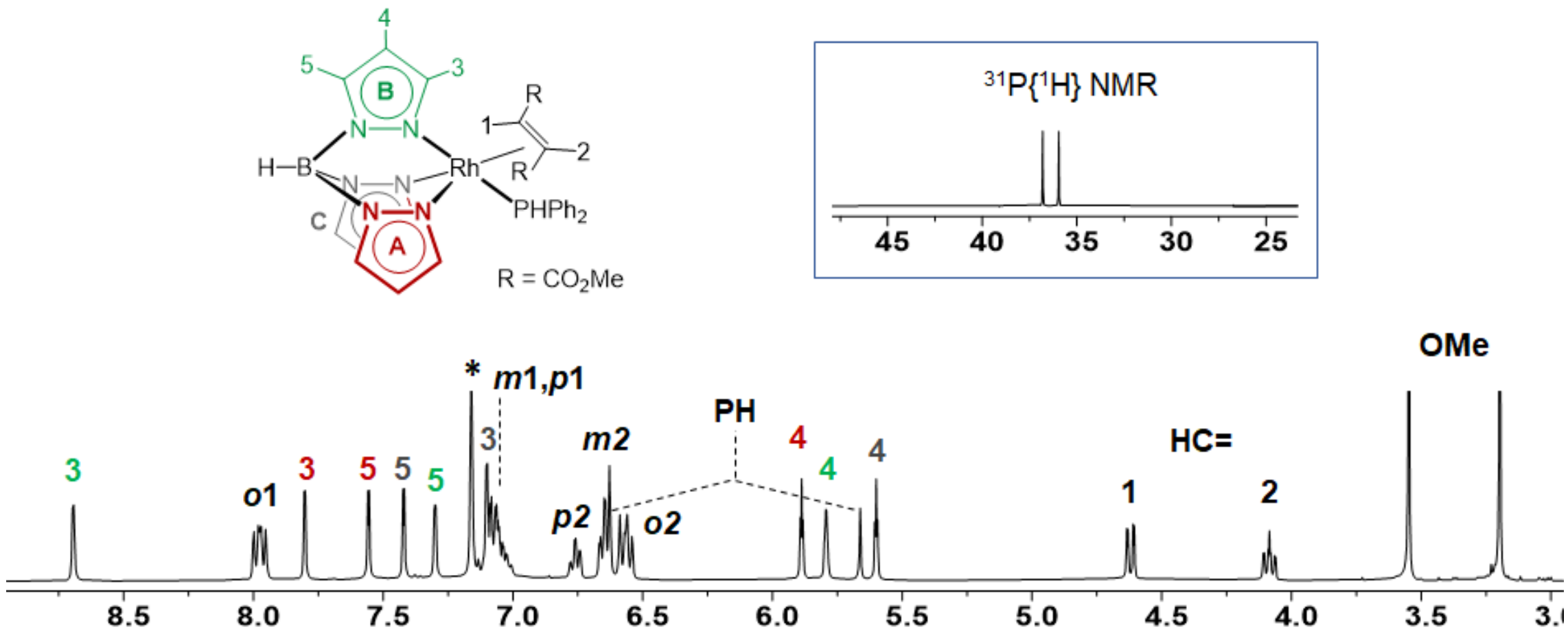


**Figure S5**. $^1H$ NMR and $^{31}P\{^1H\}$ (inset) NMR spectra of complex **8** in in $C_6D_6$. The asterisk (*) denotes the residual signal of the solvent.

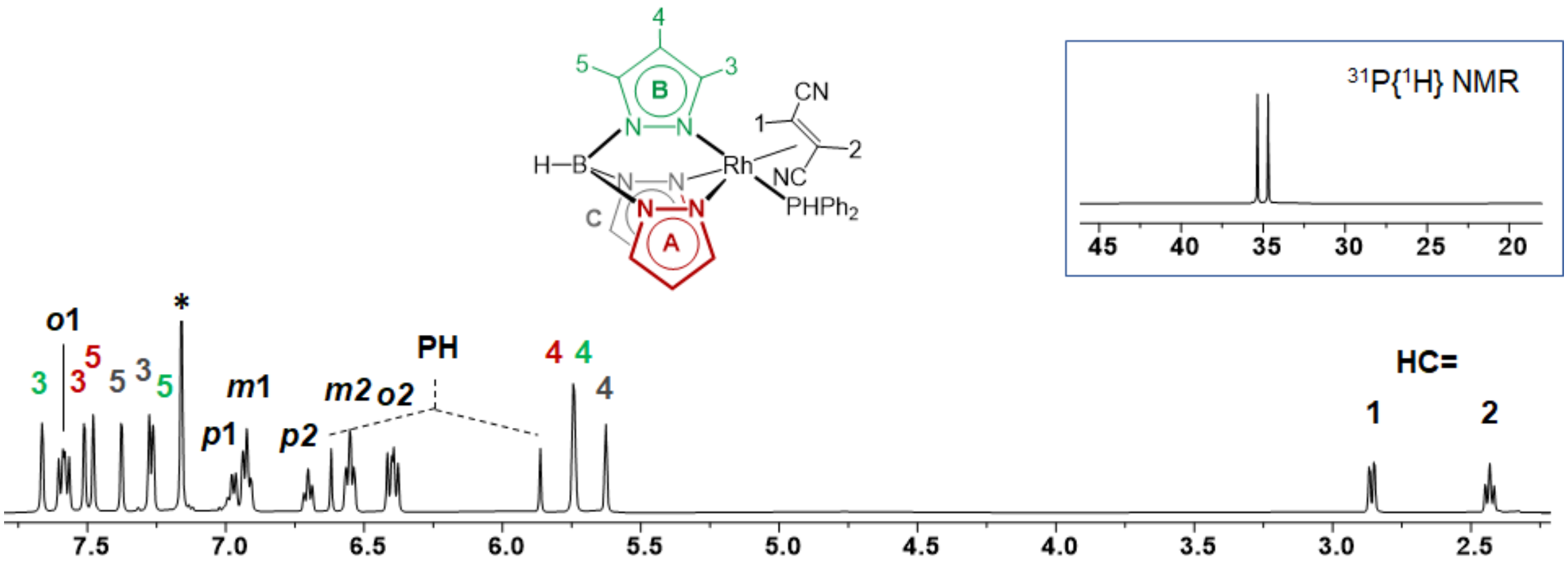


**Figure S6**. $^1H$ NMR and $^{31}P\{^1H\}$ (inset) NMR spectra of complex **9** in in $C_6D_6$. The asterisk (*) denotes the residual signal of the solvent.

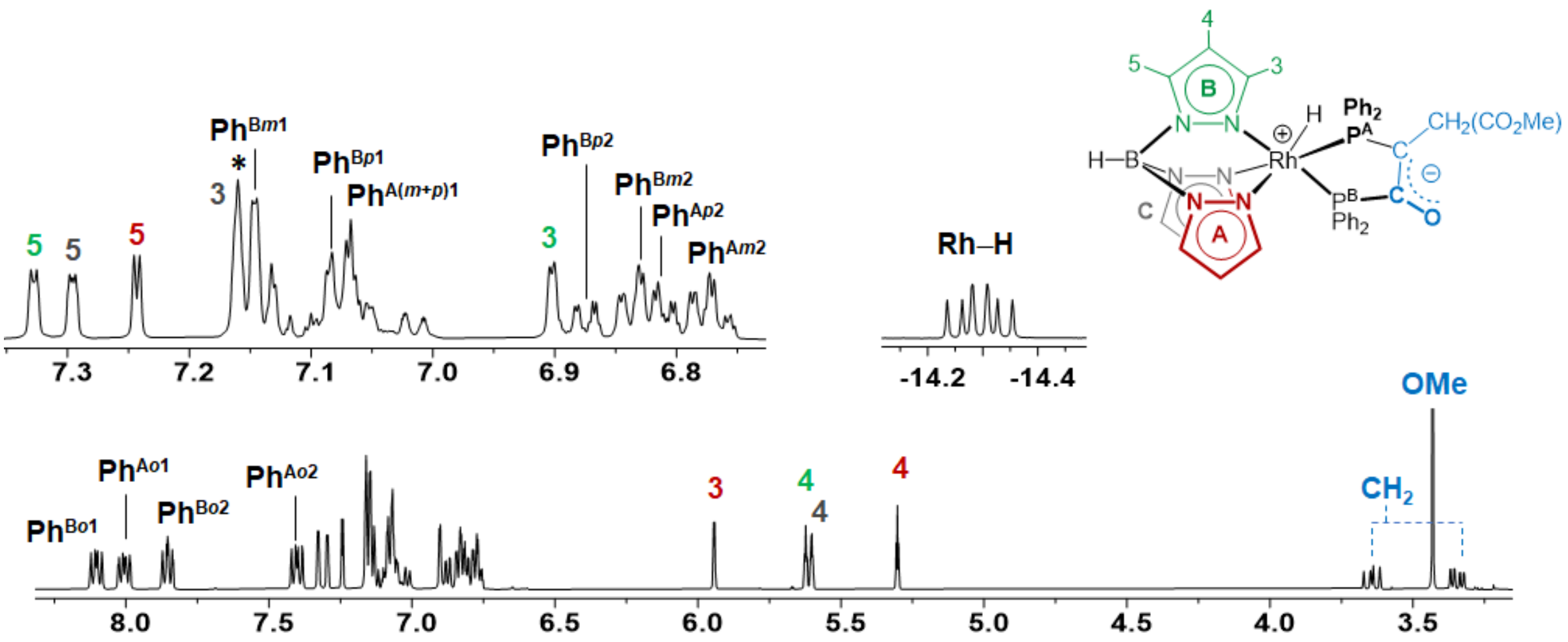


**Figure S7**. $^{1}$H NMR spectrum, and selected regions, of **11** in $C_6D_6$. The asterisk (*) denotes the residual signal of the solvent.

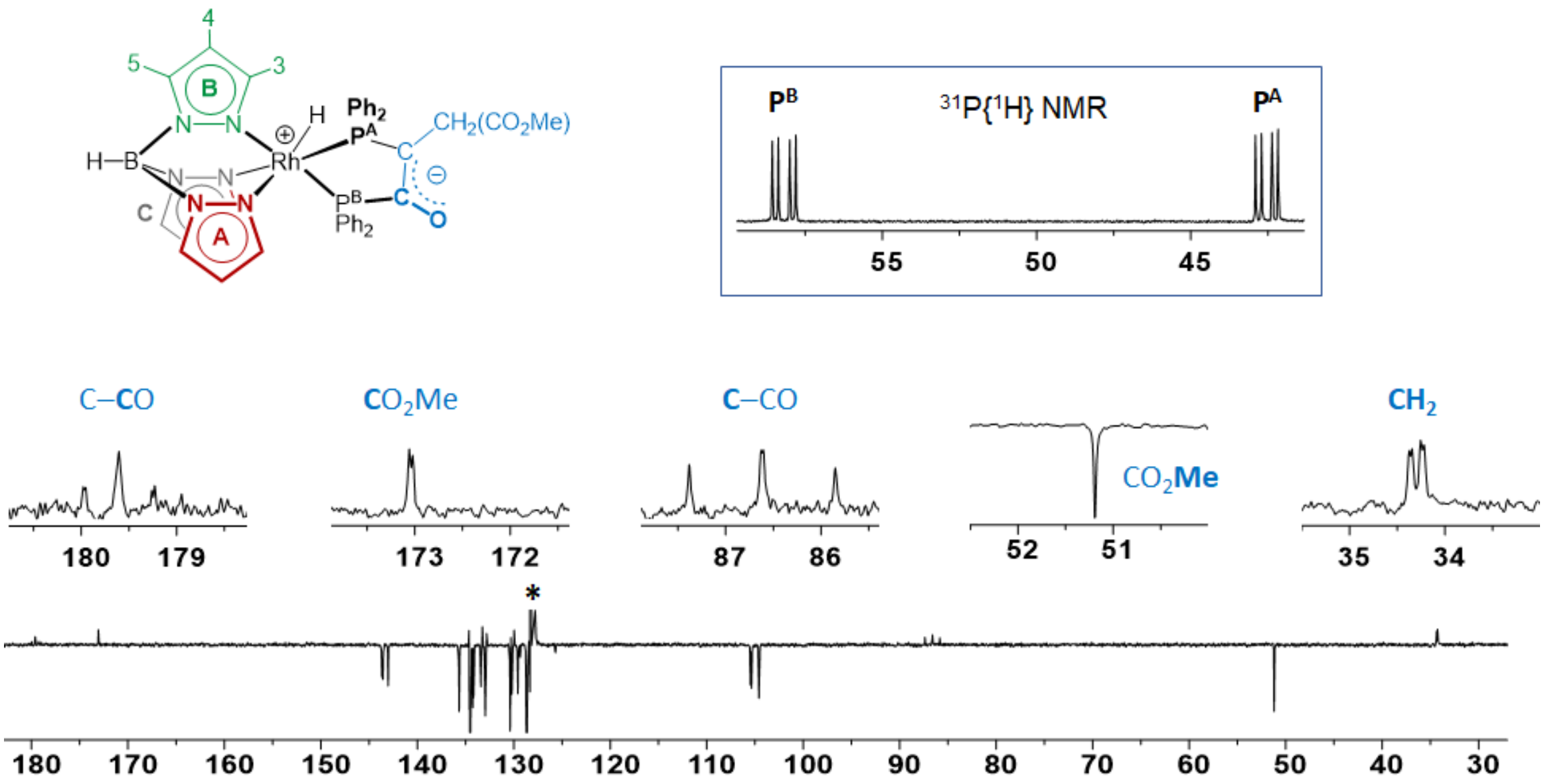


**Figure S8**. $^{13}$C{$^{1}$H}-apt NMR spectrum, and selected regions, of **11** in $C_6D_6$. The $^{31}$P{$^{1}$H} NMR spectrum is shown in the inset. The asterisk (*) denotes the residual signal of the solvent.

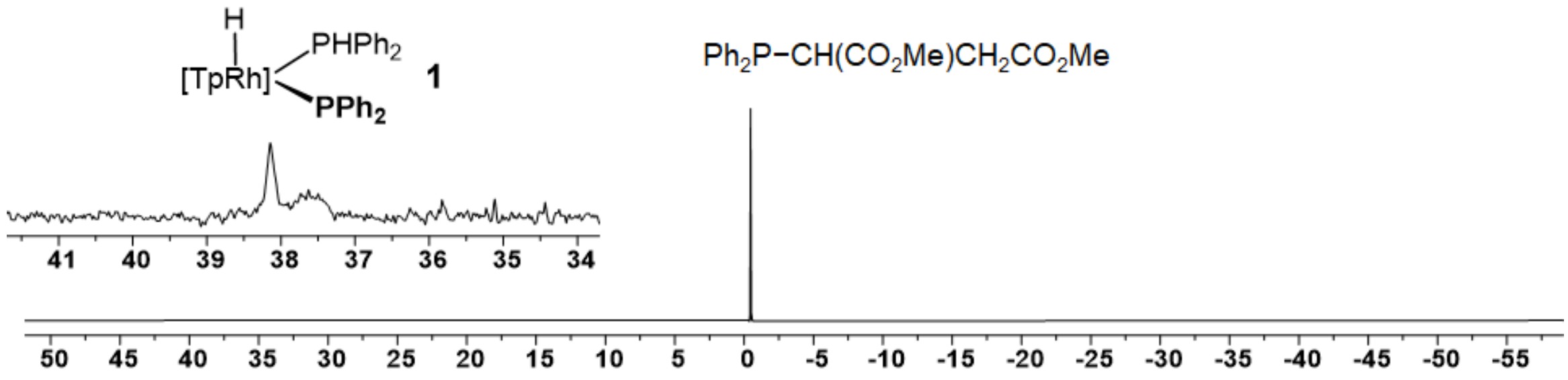


**Figure S9**. $^{31}P\{^{1}H\}$ NMR spectrum of hydrophosphanation of dimethylfumarate with $PHPh_2$ catalyzed by complex **1** at the end of the catalysis. at 25 ºC.

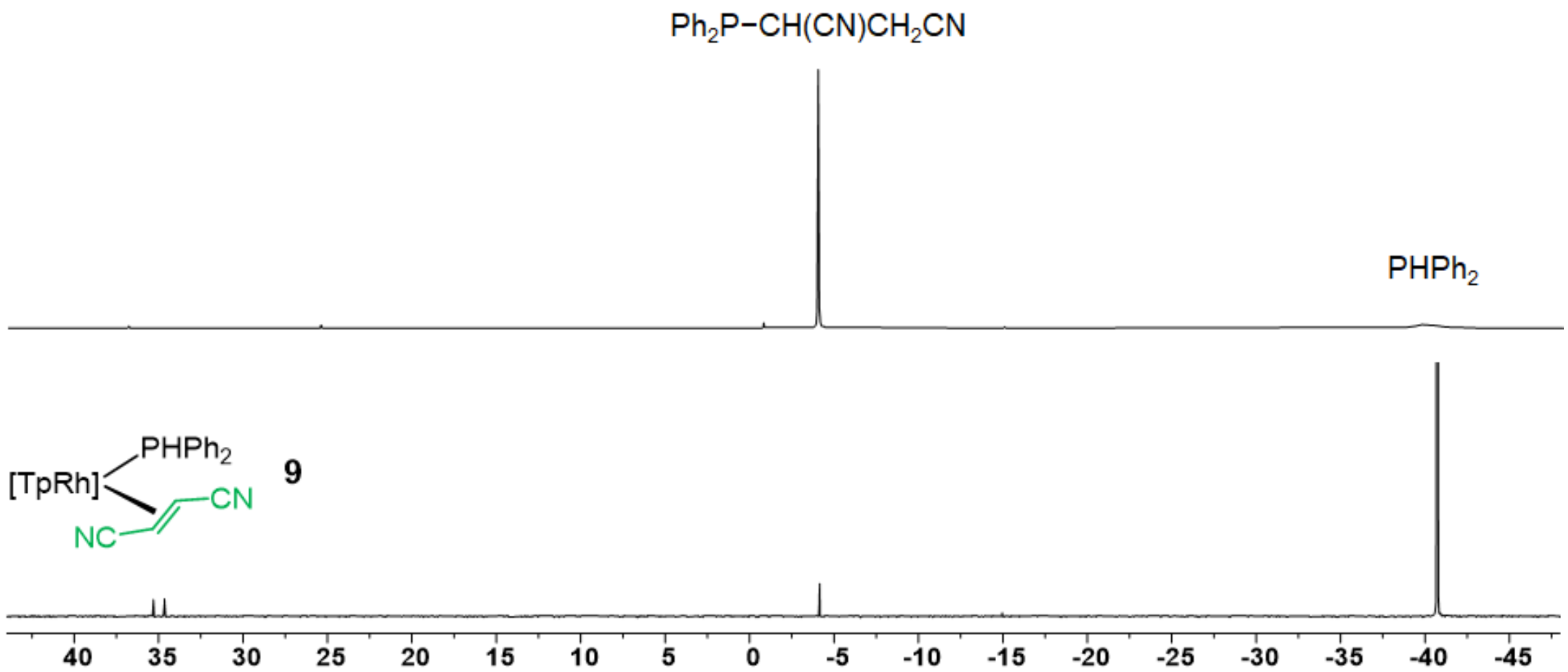


**Figure S10**. $^{31}P\{^{1}H\}$ NMR spectra of hydrophosphanation of fumaronitrile with $PHPh_2$ catalyzed by complex **1** after the first catalytic cycle (down) and at the end of the catalysis (up, around a week at 25 ºC).

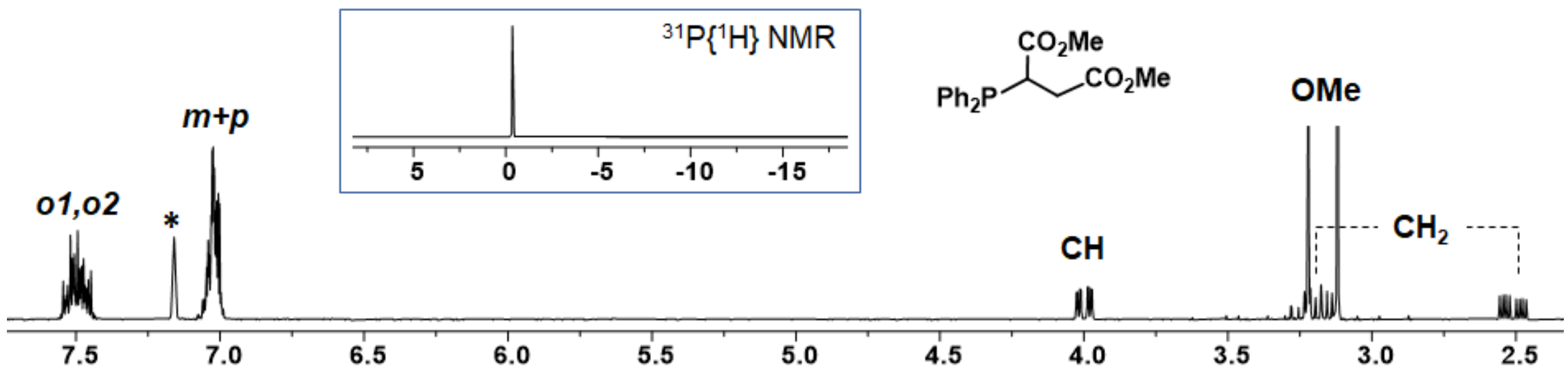


**Figure S11**. $^1$H NMR and $^{31}$P{$^1$H} (inset) NMR spectra of the phosphane $Ph_2P–CH(CO_2Me)CH_2CO_2Me$ in $C_6D_6$. The asterisk (*) denotes the residual signal of the solvent.

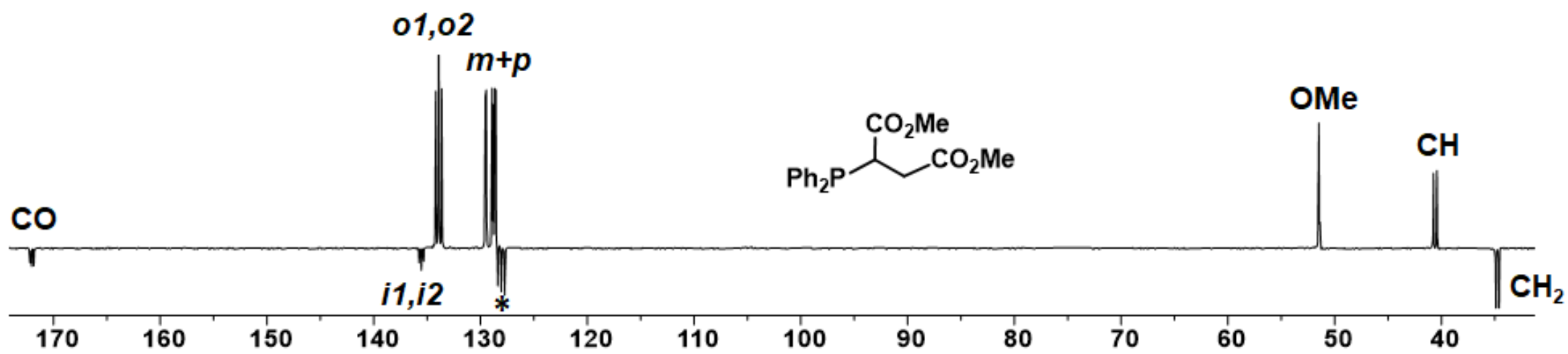


**Figure S12**. $^{13}$C{$^1$H}-apt NMR spectrum of the phosphane $Ph_2P–CH(CO_2Me)CH_2CO_2Me$ in $C_6D_6$. The asterisk (*) denotes the residual signal of the solvent.

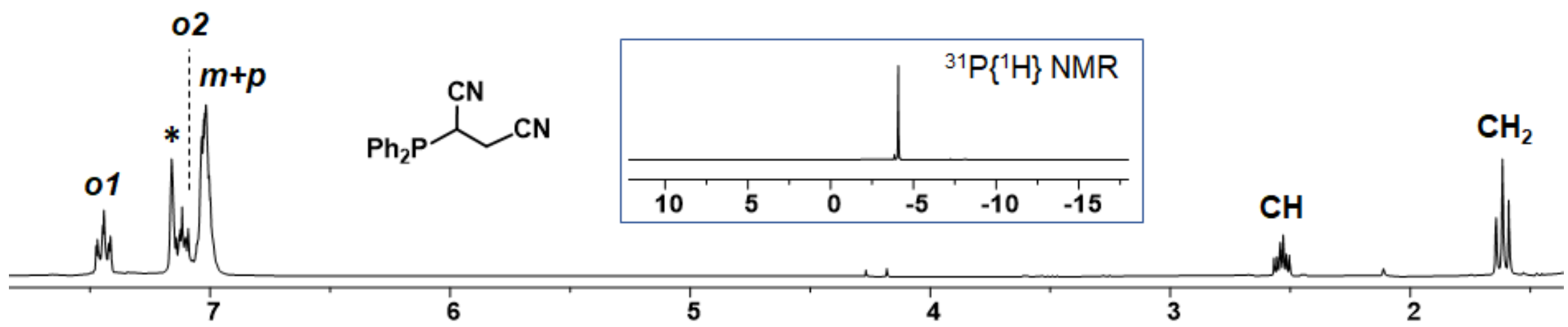


**Figure S13**. $^1$H NMR and $^{31}$P{$^1$H} (inset) NMR spectra of the phosphane $Ph_2P–CH(CN)CH_2CN$ in $C_6D_6$. The asterisk (*) denotes the residual signal of the solvent.

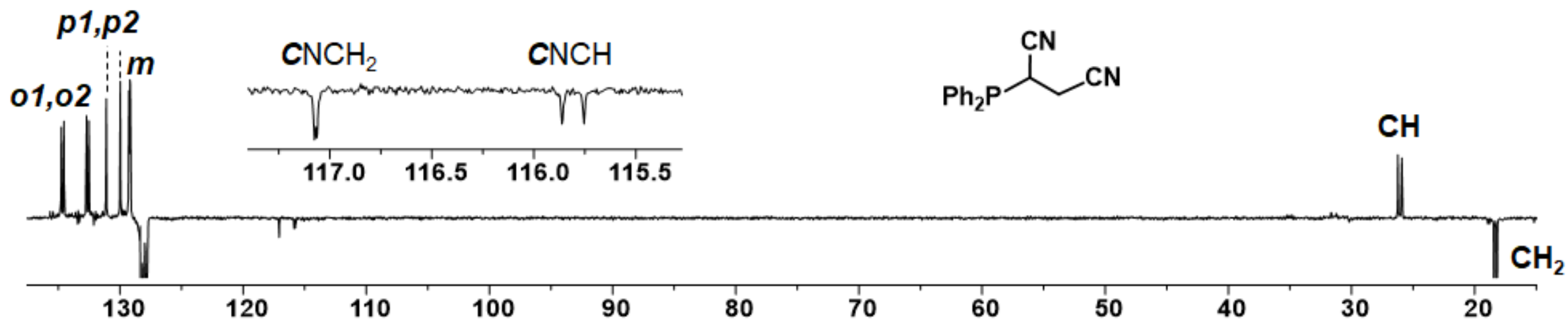


**Figure S14**. $^{13}$C{$^1$H}-apt NMR spectrum of the phosphane $Ph_2P–CH(CN)CH_2CN$ in $C_6D_6$.

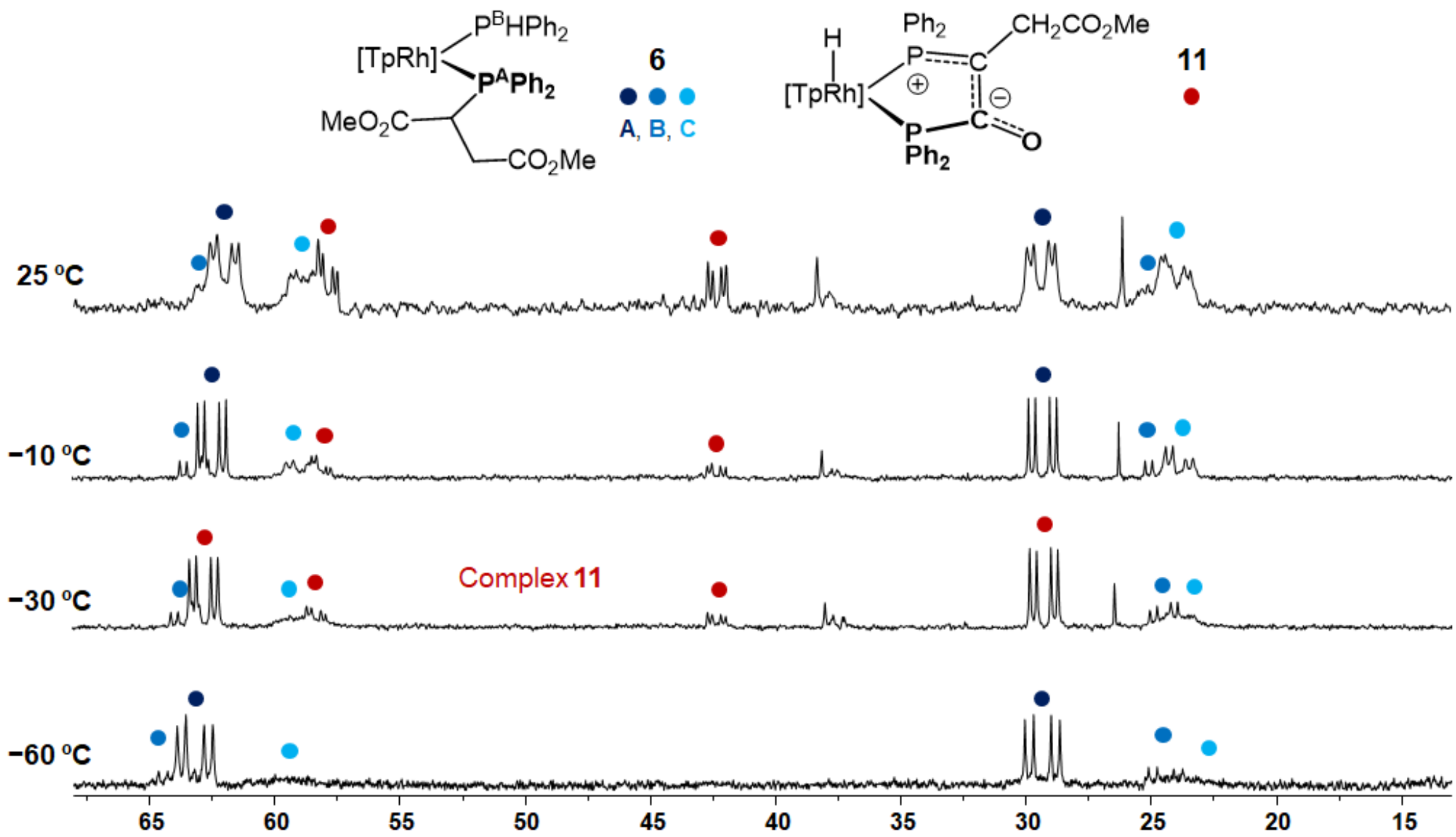


**Figure S15**. VT-$^{31}P\{^{1}H\}$ NMR of the reaction mixture of [Rh(Tp)($PHPh_2$)($C_2H_4$)] with $Ph_2P$−CH($CO_2Me$)$CH_2CO_2Me$ in toluene-$d_8$.

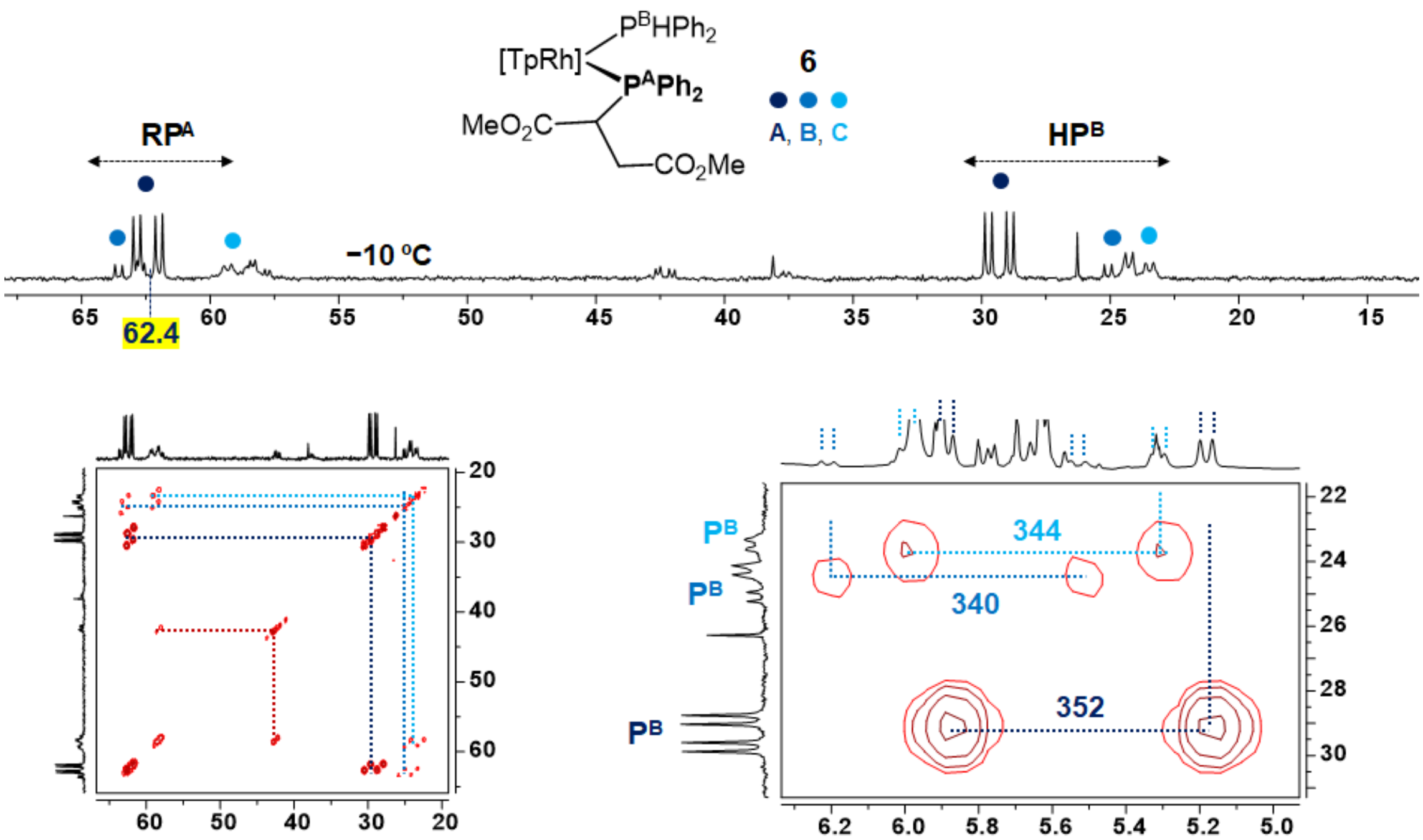


**Figure S16**. $^{31}P,^{31}P\{^{1}H\}$-cosy spectrum (down, left), selected region of the $^{1}H,^{31}P$-hmbc spectrum (down, right), and $^{31}P\{^{1}H\}$ NMR spectrum (up) of complex **6** in $d_8$-toluene at −10 ºC. Cross-peaks corresponding to complex **11** are indicated in red.

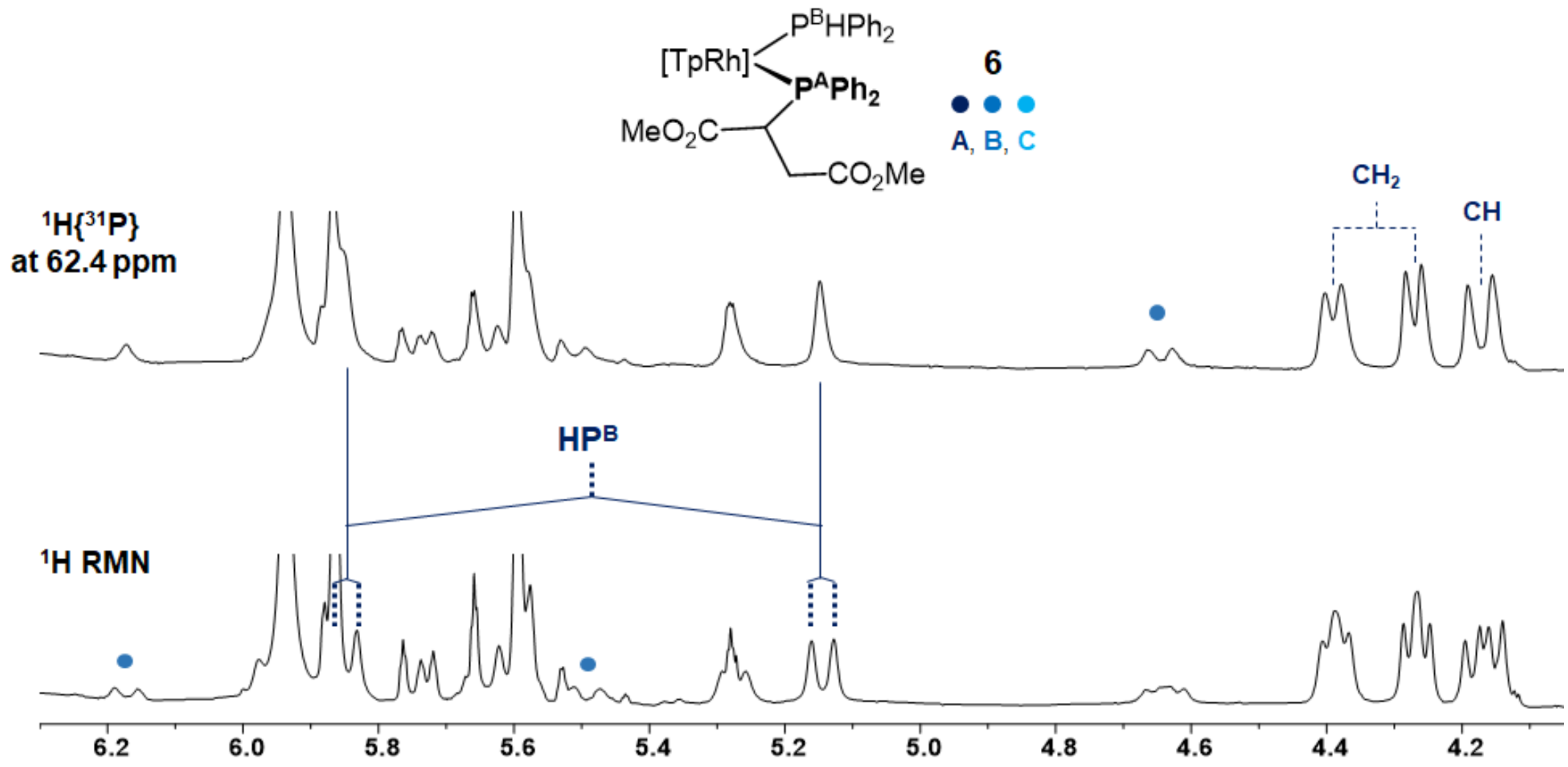


**Figure S17**. Selected region of the $^{1}H$ NMR spectrum of complex **6** in $d_8$-toluene at −10 ºC (down) and upon irradiation of the $^{31}P$ at 62.4 ppm (up).

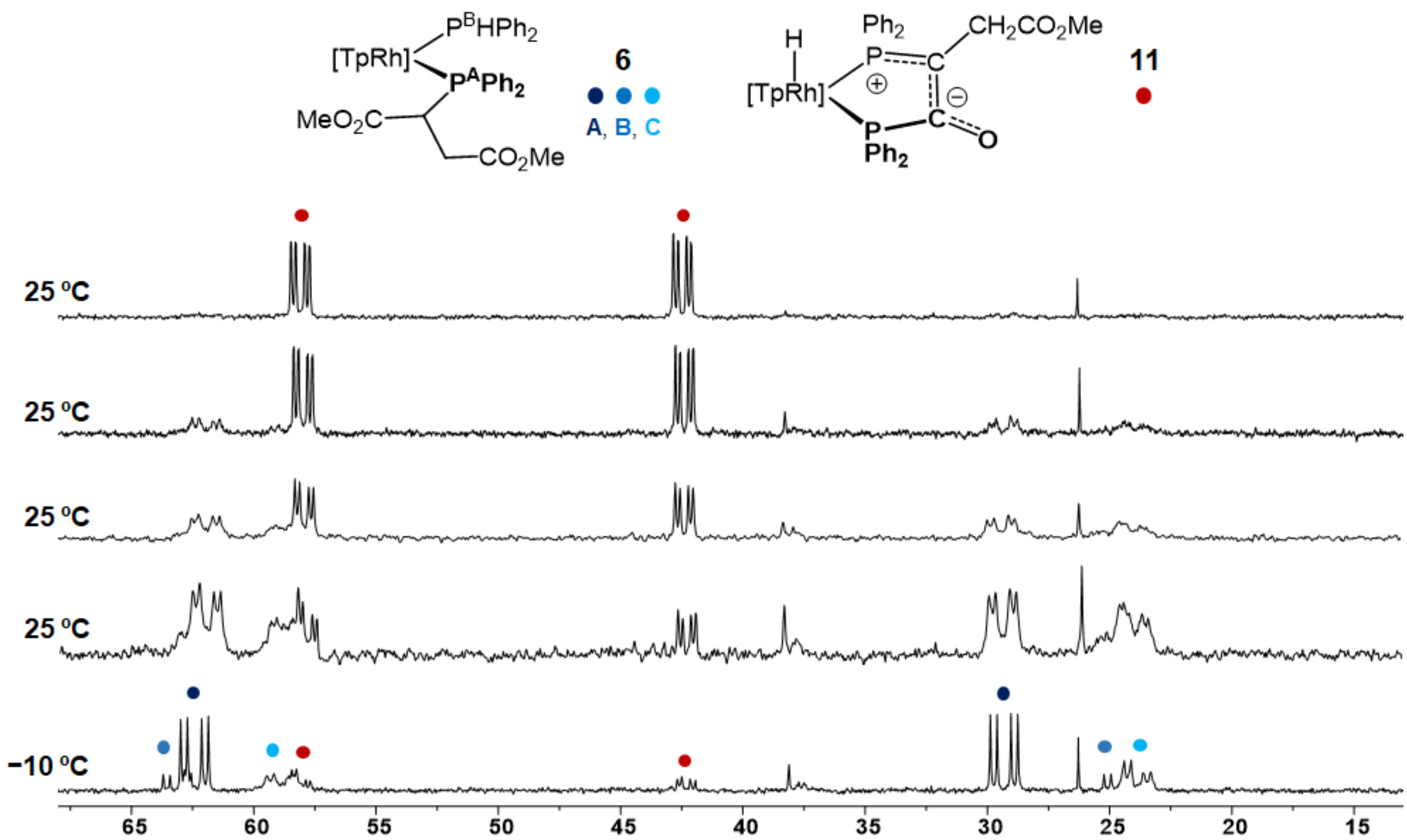


**Figure S18**. $^{31}P\{^{1}H\}$ NMR spectra corresponding to the transformation of complex **6** (species **A**, **B**, and **C**) into complex **11** over a period of 24h at 25 ºC.

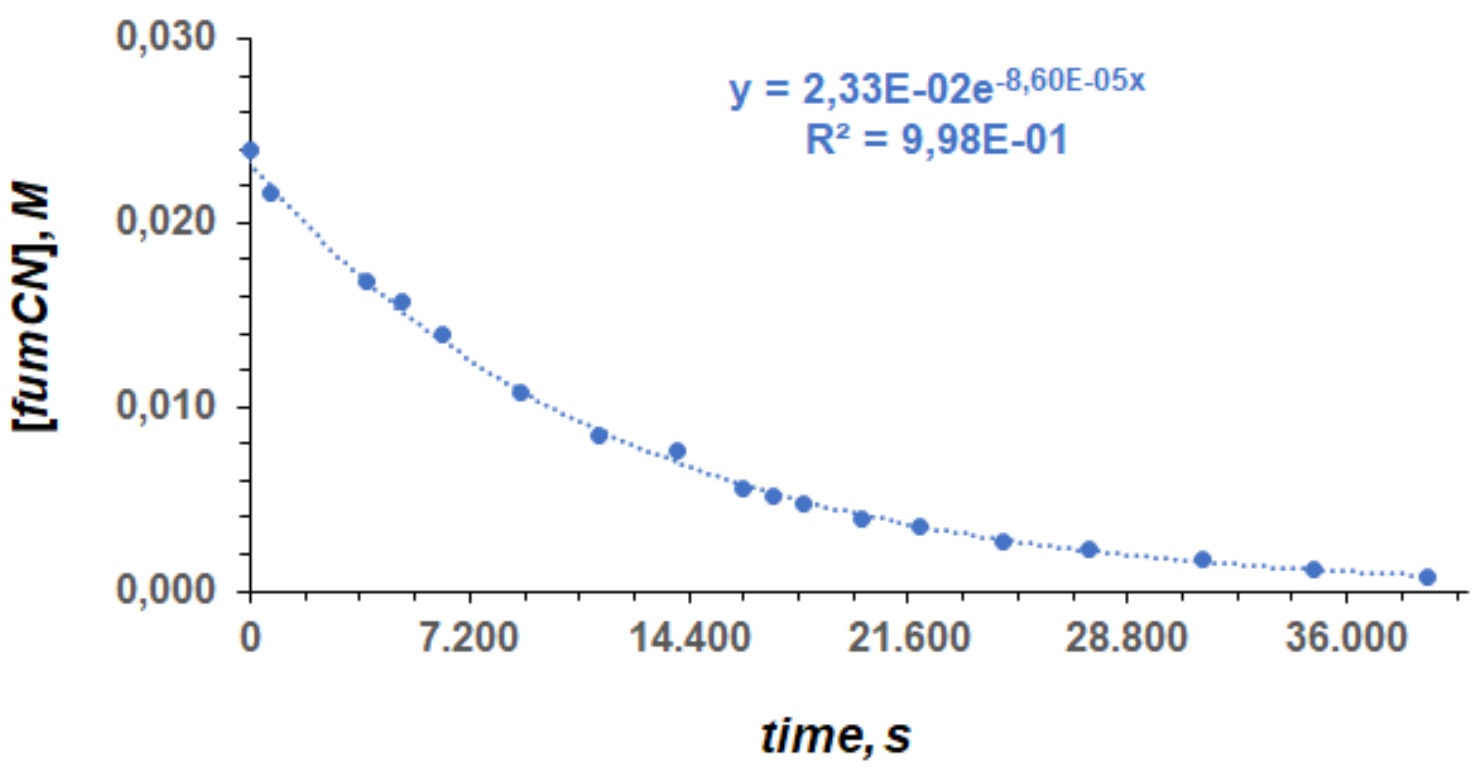


**Figure S19**. Plot of concentration of fumaronitrile (fumCN, M) *vs* time (s) for the reaction of [Rh(Tp)($PHPh_2$)(fumCN)] (**9**) with $PHPh_2$ (2.3 mol-equiv.) in $C_6D_6$ at 25 ºC.

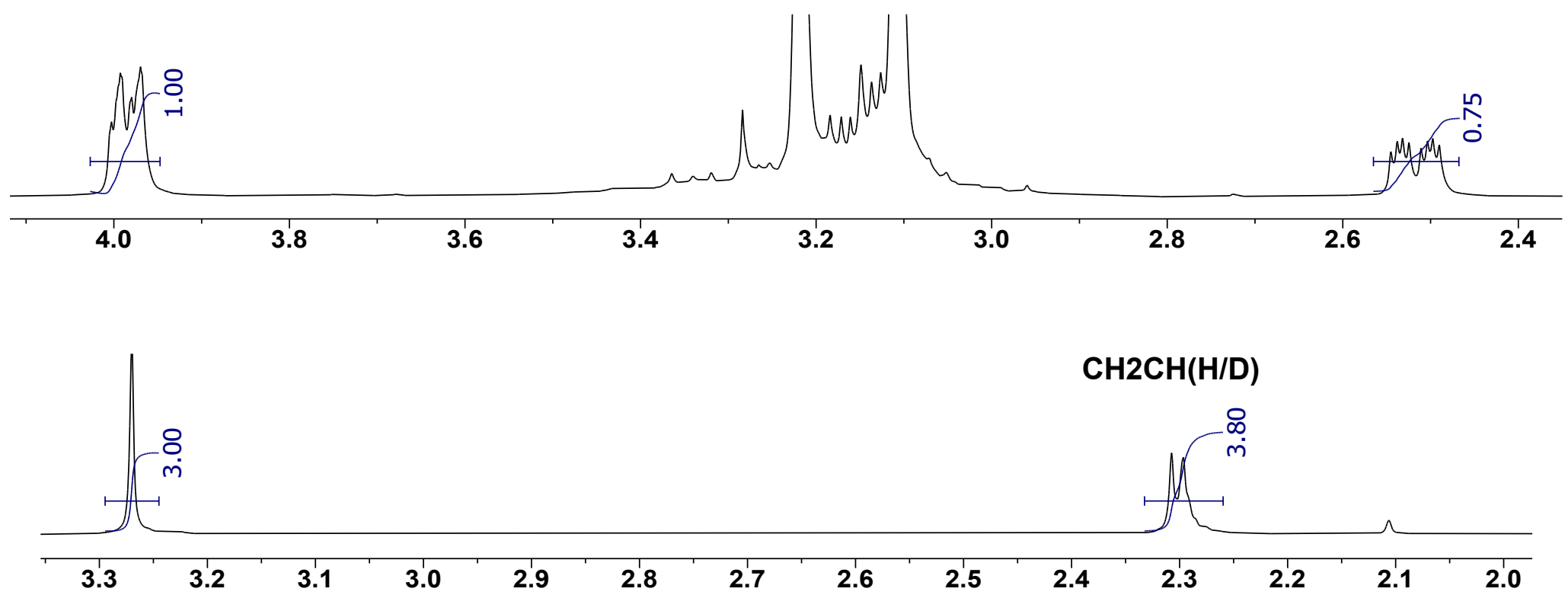


**Figure S20**. Selected region of the 1H NMR spectrum of the catalytic hydrophosphanation of fumaronitrile (top) and methylacrylate (bottom) with an equimolecular mixture of $PHPh_2$/$PDPh_2$ catalyzed by complex **1** in $C_6D_6$. Values of PH/PD = 3 and 4, respectively, were measured.

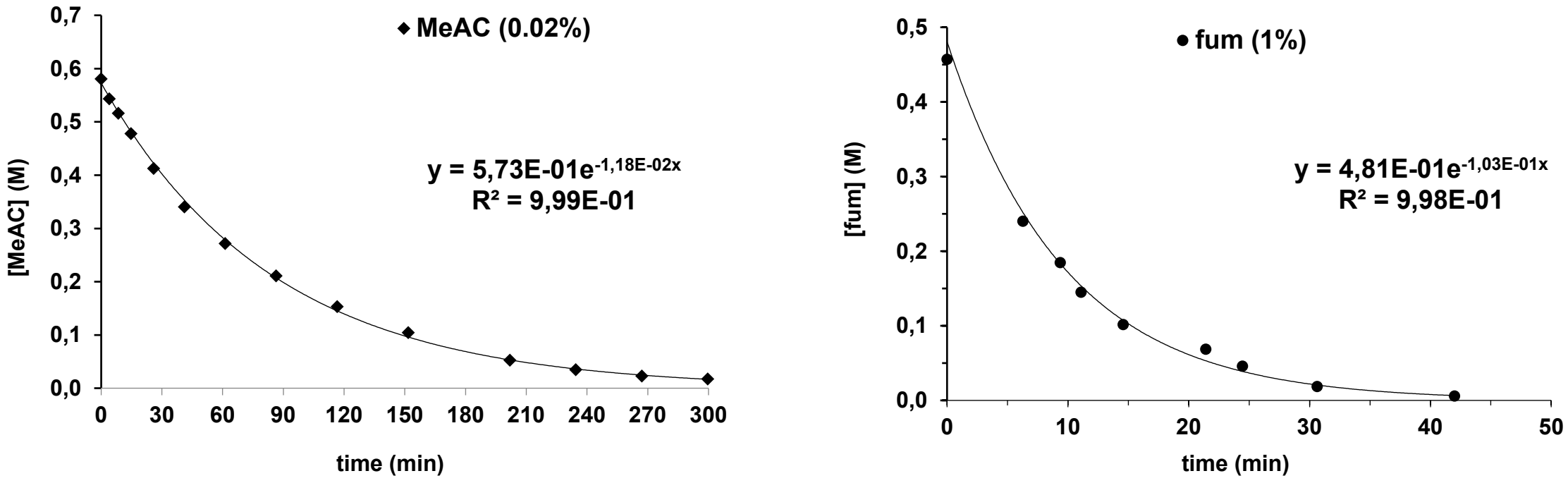


**Figure S21**. Plots of concentration of dimethylfumarate (fum, right) and methyl acrylate (MeAC, left) *vs* time (s) for olefin hydrophosphanation with $PHPh_2$, catalyzed by **1**. Reaction conditions: $PHPh_2$ (0.48 mmol), alkene (0.48 mmol), and catalyst [Rh(Tp)($PHPh_2$)(H)($PPh_2$)] (0.02% mol for MeAC and 1% mol for fum) in $C_6D_6$ ($V_t$ = 0.7 mL) at 25 °C.

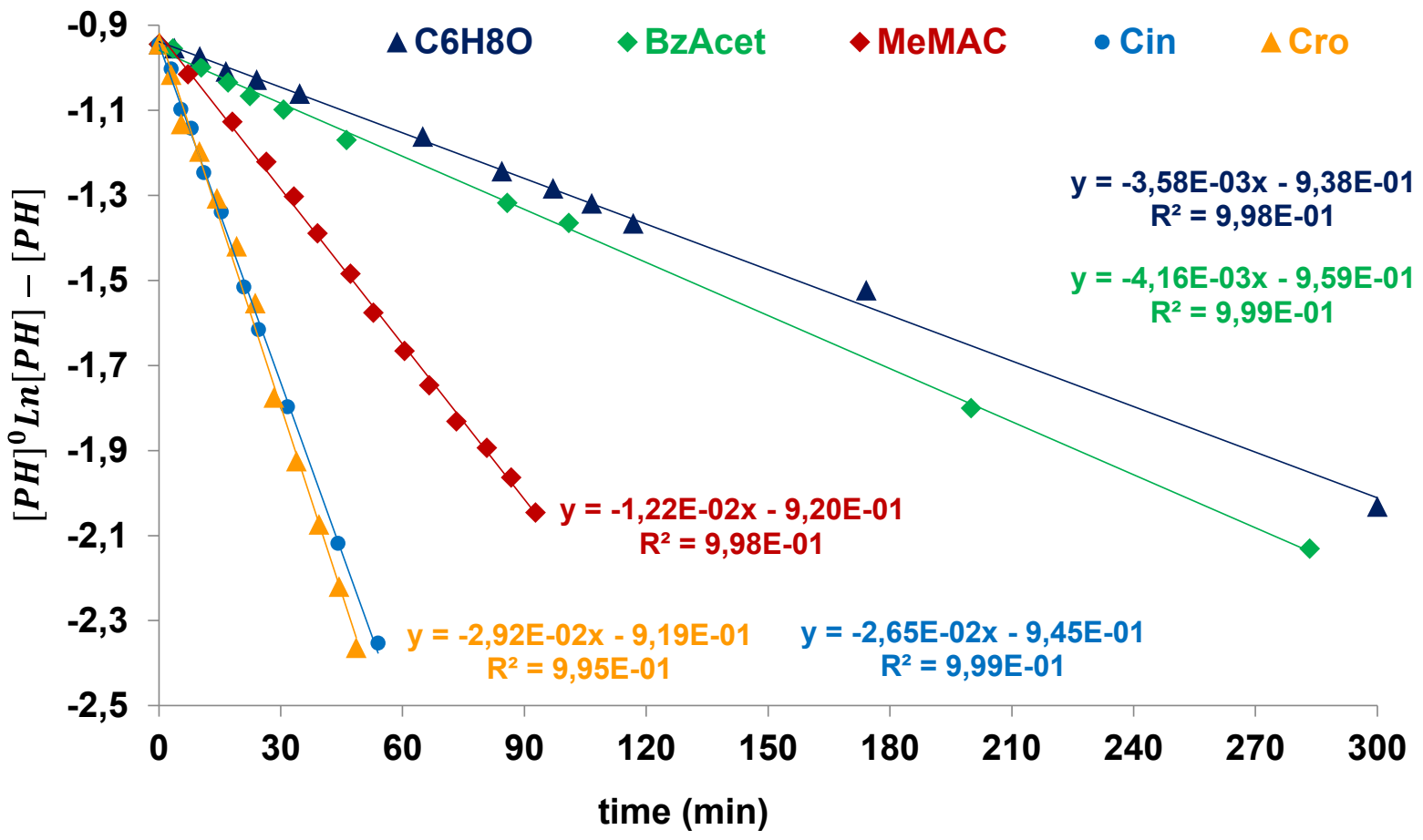


**Figure S22**. Plots of $[PHPh_2]^0 Ln([PHPh_2]) - [PHPh_2]\ (M)$ *vs* time (s) for the cyclohexenone ($C_6H_8O$), bezylidenacetone (BzAcet), methylmetacrilate (MeMAC), cinnamaldehyde (Cin), and crotonaldehyde (Cro) hydrophosphanation with $Ph_2PH$ catalyzed by **1**. Reaction conditions: $PHPh_2$ (0.48 mmol), alkene (0.48 mmol), and catalyst [Rh(Tp)($PHPh_2$)(H)($PPh_2$)] (0.024 mmol, 5% mol) in $C_6D_6$ ($V_t$ = 0.7 mL) at 25 °C.

## References


[S1] Ref. 10 in the main text

[S2] Gaussian 09 (Revision D.01): M. J. Frisch, G. W. Trucks, H. B. Schlegel, G. E. Scuseria, M. A. Robb, J. R. Cheeseman, G. Scalmani, V. Barone, B. Mennucci, G. A. Petersson, H. Nakatsuji, M. Caricato, X. Li, H. P. Hratchian, A. F. Izmaylov, J. Bloino, G. Zheng, J. L. Sonnenberg, M. Hada, M. Ehara, K. Toyota, R. Fukuda, I. J. M. Hasegawa, T. Nakajima, Y. Honda, O. Kitao, H. Nakai, T. Vreven, J. A. Montgomery Jr., J. E. Peralta, F. Ogliaro, M. Bearpark, J. J. Heyd, E. Brothers, K. N. Kudin, V. N. Staroverov, R. Kobayashi, J. Normand, K. Raghavachari, A. Rendell, J. C. Burant, S. S. Iyengar, J. Tomasi, M. Cossi, N. Rega, J. M. Millam, M. Klene, J. E. Knox, J. B. Cross, V. Bakken, C. Adamo, J. Jaramillo, R. Gomperts, R. E. Stratmann, O. Yazyev, A. J. Austin, R. Cammi, C. Pomelli, J. W. Ochterski, R. L. Martin, K. Morokuma, V. G. Zakrzewski, G. A. Voth, P. Salvador, J. J. Dannenberg, S. Dapprich, A. D. Daniels, Ö. Farkas, J. B. Foresman, J. V. Ortiz, J. Cioslowski, D. J. Fox, Gaussian, Inc., Wallingford CT, 2011.

[S3] C. Lee, W. Yang, R. G. Parr, Development of the Colic-Salvetti correlation-energy formula into a functional of the electron density, *Phys. Rev. B* **1988**, *37*, 785–789.

[S4] A. D. Becke, A new mixing of Hartree-Fock and local density-functional theories, *J. Chem. Phys.* **1993**, *98*, 1372–1377.

[S5] A. D. Becke, Density-functional thermochemistry. III. The role of exact exchange, *J. Chem. Phys.* **1993**, *98*, 5648–5652.

[S6] S. Grimme, J. Antony, S. Ehrlich, H. Krieg, A consistent and accurate ab initio parametrization of density functional dispersion correction (DFT-D) for the 94 elements, H-Pu, *J. Chem. Phys.* **2010**, *132*, 154104.

[S7] L. E. Roy, P. J. Hay, R. L. Martin, Revised Basis Sets for the LANL Effective Core Potentials, *J. Chem. Theory Comput.* **2008**, *4*, 1029–1031.
